# Comparative transcriptome analysis of isogenic cell line models and primary cancers links capicua (CIC) loss to activation of the MAPK signalling cascade

**DOI:** 10.1002/path.4894

**Published:** 2017-04-26

**Authors:** Veronique G LeBlanc, Marlo Firme, Jungeun Song, Susanna Y Chan, Min Hye Lee, Stephen Yip, Suganthi Chittaranjan, Marco A Marra

**Affiliations:** ^1^ Canada's Michael Smith Genome Sciences CentreBC Cancer AgencyVancouverBCCanada; ^2^ Genome Science and Technology ProgramUniversity of British ColumbiaVancouverBCCanada; ^3^ Department of Pathology and Laboratory MedicineUniversity of British ColumbiaBCCanada; ^4^ Department of Medical GeneticsUniversity of British ColumbiaVancouverBCCanada

**Keywords:** capicua, glioma, stomach adenocarcinoma, MAPK signalling

## Abstract

CIC encodes a transcriptional repressor, capicua (CIC), whose disrupted activity appears to be involved in several cancer types, including type I low‐grade gliomas (LGGs) and stomach adenocarcinomas (STADs). To explore human CIC's transcriptional network in an isogenic background, we developed novel isogenic CIC knockout cell lines as model systems, and used these in transcriptome analyses to study the consequences of CIC loss. We also compared our results with analyses of transcriptome data from TCGA for type I LGGs and STADs. We identified 39 candidate targets of CIC transcriptional regulation, and confirmed seven of these as direct targets. We showed that, although many CIC targets appear to be context‐specific, the effects of CIC loss converge on the dysregulation of similar biological processes in different cancer types. For example, we found that CIC deficiency was associated with disruptions in the expression of genes involved in cell–cell adhesion, and in the development of several cell and tissue types. We also showed that loss of CIC leads to overexpression of downstream members of the mitogen‐activated protein kinase (MAPK) signalling cascade, indicating that CIC deficiency may present a novel mechanism for activation of this oncogenic pathway. © 2017 The Authors. Journal of Pathology published by John Wiley & Sons Ltd on behalf of Pathological Society of Great Britain and Ireland.

## Introduction

Low‐grade gliomas (LGGs) can be separated into three major molecular subtypes that provide superior prognostic information compared to traditional histological classification: type I (*IDH1/2* mutated and 1p/19q co‐deleted), type II (*IDH1/2* mutated), and type III (*IDH1/2* wild type) 1, 2, 3, 4. Type I LGGs, which are strongly associated with oligodendrogliomas, are of particular interest because they are associated with better survival, slow growth, and increased chemosensitivity 1. Hemizygous mutations in the capicua (*CIC*) gene, located on chromosome 19q13.2, are found in ∼50–70% of type I LGGs, but are absent from other glioma subtypes 5, 6, 7, 8. Recent studies have indicated that *CIC* mutations are associated with poorer outcome for type I LGGs 9, 10. Multiple distinct *CIC* mutations have also been found within different regions of single lesions 1, indicating that multiple, independently arising *CIC* mutations may contribute to the progression of a single tumour. Together, these observations are compatible with the notion that *CIC* mutations contribute to oncogenic progression in type I LGGs.

CIC was originally identified in *Drosophila melanogaster* as a tissue‐specific transcriptional repressor involved in developmental regulation 11, 12, 13. CIC homologues found across metazoans share at least two highly conserved domains: a high mobility group (HMG) box domain involved in DNA binding, and a C‐terminal domain (C1) that appears to be necessary for repression in certain contexts in *Drosophila* 14, 15, 16, 17. CIC is a transducer of receptor tyrosine kinase (RTK) signalling that functions through default repression; upon RTK activation, CIC is directly phosphorylated by extracellular signal‐regulated kinase (ERK) 11, 18, leading to inhibition of CIC activity and de‐repression of its target genes. In humans, CIC's most well‐characterized target genes are those encoding the oncogenic transcription factors ETV1, ETV4, and ETV5 19, 20, 21, which have been implicated in several cancer types 22, 23, 24.

In this study, we used integrative bioinformatics approaches and novel isogenic cell line models to explore human CIC's transcriptional network. We identified novel candidate targets of CIC regulation, and confirmed some of these as direct targets. We showed that, while CIC appears to have some context‐specific activity, CIC deficiency is associated with disruption of similar pathways and processes in biologically distinct contexts, including disruption of cell adhesion‐related processes and aberrant overexpression of the mitogen‐activated protein kinase (MAPK) signalling cascade.

## Materials and methods

### Cell culture, cell lysate preparations, and western blot analysis

HEK293a, HOG, and immortalized normal human astrocytes (NHA) cell lines were cultured in Dulbecco's modified Eagle's medium supplemented with 10% (v/v) heat‐inactivated fetal bovine serum (Life Technologies, Ottawa, Ontario, Canada). Cell culture was performed in a humidified, 37 °C, 5% CO_2_ incubator. Cell lysate preparations and western blot analyses were performed according to standard protocols, which are described in detail in supplementary material, Supplementary materials and methods. Antibody and primer information can be found in supplementary material, Table S1.

### Microarray expression profiling

The following biological replicates were analysed: three HEK‐derived *CIC* wild type (*CIC* ^WT^) lines (HEK, F12, and B7) and three HEK‐derived *CIC* knockout (*CIC* ^KO^) lines (D10, A9, and D1); and three separate passages each of the parental *CIC* ^WT^ (HOG) line and of the HOG‐derived *CIC* ^KO^ (F11) line. RNA extraction was performed with the RNeasy Plus Mini Kit (Qiagen, Montreal, Quebec, Canada), according to the manufacturer's recommendations. Microarray expression profiling was performed with the GeneChip Human Gene 2.0 ST array (Affymetrix, Santa Clara, CA, USA) at the Centre for Applied Genomics, The Hospital for Sick Children, Toronto, Canada. Robust multichip average (RMA) normalization was performed with the R/Bioconductor package oligo 25 (version 1.34.2), with gene‐level summarization of core probeset data. Annotation was performed with the R/Bioconductor package hugene20sttranscriptcluster.db (version 8.5.0), and only transcript clusters that mapped to single genes were retained for further analyses. Multiple transcript clusters that mapped to identical genes were aggregated by the use of median expression values. To identify candidate target genes, fold‐change (FC) differences in gene expression were calculated for each gene between each individual *CIC* ^KO^/*CIC* ^WT^ pair. Genes with an FC value of >1.5 in at least four (HEK) or six (HOG) comparisons were considered to be differentially expressed (DE) 26. The data are accessible through the Gene Expression Omnibus (dataset GSE80359).

### TCGA expression analyses

RNA‐sequencing results were obtained from the TCGA data portal (https://tcga‐data.nci.nih.gov/tcga/; see supplementary material, Table S2, for sample information). Motivated by our observation that a proportion of *CIC* ^WT^ samples in type I LGGs showed relatively low *CIC* mRNA expression (supplementary material, Figure S1), and given the possibility that alterations other than sequence variants could affect *CIC* expression 27, we analysed data from *CIC* ^WT^ samples with *CIC* expression greater than the first quartile, giving a total of 68 *CIC* ^WT^ samples and 39 samples with truncating *CIC* mutations. For stomach adenocarcinoma (STAD), samples with a *CIC* copy number loss (*CIC* ^loss^, *n* = 48) were compared to samples with intact *CIC* (*n* = 155). Samples with a *CIC* mutation were excluded. The R/Bioconductor package DESeq2 28 (version 1.10.0) was used to conduct differential expression analyses.

## Results

### Transcriptome analysis of CIC ^KO^ cell line models identifies known and novel candidate targets of CIC transcriptional regulation

**Figure 1:**
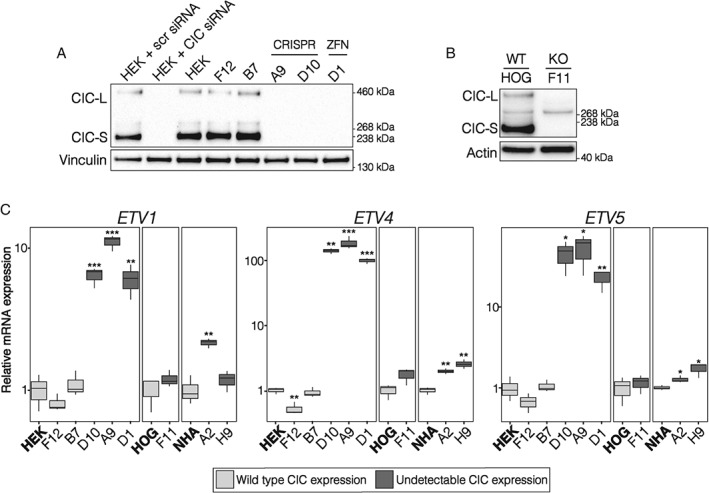
Novel CIC ^KO^ cell line models lack functional CIC. (A) Representative western blot of HEK‐derived CIC ^WT^ (HEK, F12, and B7) and CIC ^KO^ (A9, D10, and D1) cell lines profiled by the use of microarrays. A9 and D10 were obtained using the CRISPR/Cas9 technology, and D1 was obtained using a zinc finger nuclease. HEK + siRNA: HEK293a cells treated with a ‘scrambled’ non‐targeting control (scr) or CIC‐specific siRNA to confirm CIC antibody specificity. Vinculin was used as a loading control. (B) Representative western blot of the HOG cell line and its CIC ^KO^ derivative (F11). Actin was used as a loading control. (C) Tukey boxplots showing relative ETV1/4/5 mRNA expression, as measured by reverse transcription (RT)‐qPCR, in the indicated cell lines compared to their respective parental cell line (in bold). Data were obtained from three independent experiments. *p < 0.05, **p < 0.01 and ***p < 0.001 relative to the parental cell line (two‐sided Student's t‐test).

In an effort to minimize the confounding effects of the multiple mutations found in cancer genomes and their impacts on the transcriptome, we generated isogenic *CIC* ^KO^ cell lines by using a zinc finger nuclease 29 and the CRISPR/Cas9 30, 31 technology in HEK293a (HEK) and glioma‐derived HOG cells 32 (supplementary material, Figure S2A). Both approaches were designed to produce insertions or deletions within exon 2, which is shared between the short (CIC‐S) and long (CIC‐L) CIC isoforms 33 (supplementary material, Figure S2B). Three HEK‐derived monoclonal cell lines and one HOG‐derived monoclonal cell line with undetectable CIC expression were obtained (Figure 1A, B; supplementary material, Figure S2C). We functionally validated the *CIC* ^KO^ lines by measuring the expression of the known direct CIC targets *ETV1*, *ETV4*, and *ETV5* 19, 20, 21. The HEK‐derived *CIC* ^KO^ lines had significant increases in *ETV1*/*4*/*5* expression relative to the *CIC* ^WT^ controls, and the HOG‐derived *CIC* ^KO^ line showed similar trends, particularly for *ETV4* (Figure 1C). Together, the lack of detectable CIC protein expression and the increased expression of known CIC targets indicated that our *CIC* ^KO^ lines had lost CIC's transcriptionally repressive function.

We next performed microarray gene expression analyses on our cell line models to identify genes whose expression was affected by CIC loss (supplementary material, Tables S3 and S4). Interestingly, although CIC has been observed to function as a transcriptional repressor, our list of candidate targets obtained from the HEK dataset was approximately equally distributed between genes that showed overexpression (427 of 929 genes, 46%) and underexpression (502 of 929 genes, 54%) in *CIC* ^KO^ lines (supplementary material, Table S3). Of note, the HOG dataset showed a more skewed distribution, with 411 of 611 genes (67%) showing higher expression in the *CIC* ^KO^ line (supplementary material, Table S4). While the HEK‐derived *CIC* ^KO^ lines showed increased expression of the known CIC targets *ETV1*, *ETV4*, and *ETV5*, only *ETV4* passed the threshold for increased expression in the HOG‐derived *CIC* ^KO^ lines, although *ETV5* showed a similar trend. Together, these results indicate that CIC may have some context‐dependent targets and/or activity. Interestingly, previous studies that have compared transcriptome profiles of type I LGGs have identified either a majority of downregulated genes (66%) 10 or exclusively upregulated genes 34 in *CIC* mutant samples (Table 1) 35, 36.

**Table 1:** Overlap with previously identified candidate targets of CIC transcriptional regulation

	**Glioma** 10, 34	**Lung cancer cell lines** 35	**Prostate cancer cell lines** 36
HEK	***CCND1***, ***DUSP4***, ***DUSP6***, ***ETV1***, ***ETV4***, ***ETV5***, *FLRT3*, ***GPR3***, *HPCAL4*, *OLIG2*, *PLPPR5*, *PPP1R14C*, ***PPP2RC2***, ***ROBO2***, ***SHC3***, ***SLC35F1***, *SOX11*, ***SPRED1***, ***SPRED2***, ***SPRY4***, ***TMOD1***, ***TRAPPC9***	*CKMT1A*, ***ETV4***, ***ETV5***, *KRT19*, *MYO10*, ***PRAME***, ***PTPN9***, ***SPRED1***, *SERPINB9*, *SOX4*, ***ZNF486***	***CRABP1***, ***CTGF***, *IFI44L*, *LINC01116*, *MIR570*, ***PPP2R2C***, ***TPD52L1***, *VCAN*, *VTRNA1‐2*, *ZNF702P*
HOG	*C3orf80*, ***DUSP6***, ***ETV4***, ***GPR3***, *ICA1*, *LRRC4C*, ***NRG1***, ***RAB31***, ***ROBO2***, ***SPRY4***, *STAMBPL1*, *STC2*	*COTL1*, ***CRABP2***, *DZIP3*, ***ETV4***, ***FAS***, ***NRG1***, *NUAK2*, ***NUDT7***, *PPARG*, *PRTFDC1*, *SULT2B1*, *TCEAL1*, *TBL1X*	***ADAMTS1***, *BHLHE41*, *CCDC15*, ***COL8A1***, ***CRABP2***, ***CTGF***, *EPGN*, *GMPR*, *IL22RA1*, ***MOXD1***, *NPY1R*, *PKIB*, ***RAB31***, *SNAI2*, *TBC1D1*, ***TMEM171***, ***TPD52L1***, *UCP2*
Type I LGG	*ANKRD55*, *BAALC*, *BCL2*, ***BACH2***, *C2orf27A*, *C3orf31*, *C6orf118*, *C8orf56*, *CADPS*, *CAMK2N1*, ***CCND1***, *CD82*, ***CNTNAP4***, ***CREB3L1***, *DIAPH2*, ***DCLK1***, *DLL3*, ***DUSP4***, ***DUSP6***, *ELFN1*, *EPN2*, *ESRRG*, *ETS1*, ***ETV1***, ***ETV4***, ***ETV5***, *FBFBP3*, *FGFR1*, *FOXP4*, *GCNT2*, *GFRA1*, ***GLDC***, *GLT25D2*, ***GPR3***, ***IPO8***, *KCNIP1*, *KCNK3*, *KIAA1598*, *LASP1*, *LBH*, ***LMO1***, ***LOC92659***, *LPPR5*, *MGC12982*, *NCAN*, *NLGN3*, *NPPA*, ***NRG1***, *NUDT9P1*, *PEX5L*, ***PDE4B***, *PDGFRA*, ***RAB31***, *RASGRF1*, *RNF216L*, ***SCARA5***, *SEMA4D*, *SIX1*, *SCEL*, ***SHC3***, *SPOCK3*, *SLC29A1*, ***SLC35F1***, ***SPRED1***, ***SPRED2***, ***SPRY4***, *SPSB4*, *TACC2*, *TMC3*, *TMEM158*, ***TMOD1***, *TRAF4*, ***TRAPPC9***, *TRIB2*, *TTLL7*, *UHRF1*, *VSIG10*, *WSCD1*, *ZBTB8B*, *ZSWIM4*	*CNP*, ***ETV4***, ***ETV5***, *HAS3*, *HEXIM2*, *ID4*, ***IPO8***, *LPGAT1*, ***NRG1***, *NRTN*, ***NUDT7***, *PAIP2B*, ***PDE4B***, ***PTPN9***, *SKAP2*, ***SPRED1***, *TM4SF18*, *YWHAQ*	***CRABP1***, ***CREB3L1***, *GPR4*, *LRIG1*, *MARCH9*, ***MOXD1***, *MT1G*, *MT1L*, *PLA2G1B*, *PPL*, *PRPH*, ***RAB31***, *ROBO4*, ***SCARA5***, ***TMEM171***, ***TPD52L1***
High‐confidence candidate targets	***CCND1***, ***DUSP4***, ***DUSP6***, ***ETV1***, ***ETV4***, ***ETV5***, ***PLPPR5***, ***RAB31***, ***SHC3***, ***SPRED1***, ***SPRED2***, ***SPRY4***, ***TRAPPC9***	***NUDT7***, ***PTPN9***, ***SPRED1***	***CRABP1***, ***RAB31***, ***TPD52L1***
STAD	*ADAMTS2*, *ALK*, ***BACH2***, *BAALC*, *BCL2*, *BMPER*, ***CNTNAP4***, ***DCLK1***, *DPP6*, ***ETV4***, *FAM65B*, *FKBP5*, ***GLDC***, *GPR17*, *ISM1*, *KCND2*, *KLF9*, ***LMO1***, ***LOC92659***, *LRRC7*, *NRXN2*, *NXPH3*, ***PDE4B***, ***SCARA5***, *SFRP1*, *SHROOM2*, *SNCAIP*, *TMEM132C*, ***TMOD1***	*ATP2B4*, *C11orf86*, *CREB3L3*, *CRISPLD2*, *DPYSL3*, ***ETV4***, ***FAS***, *HEYL*, ***PDE4B***, ***PRAME***, *PRX*, *TGFB3*, *S100A9*, *ZCCHC24*, *ZNF217*, ***ZNF486***, *ZNF772*	*ADAM12*, ***ADAMTS1***, *AK5*, *ARHGDIB*, *C1R*, *C1S*, *COL6A3*, ***COL8A1***, ***CRABP1***, *FAM107A*, *GAS6*, *GHRL*, *GLI3*, *HCLS1*, *HIST1H2BH*, *HLF*, *LCP1*, ***MOXD1***, *OPRL1*, *PLEKHO1*, *PRPH*, *RUNDC3B*, *SCARA5*, *SERPINB2*, *TGFB1*

LGG, low‐grade glioma; STAD, stomach adenocarcinoma.

The genes identified in this study (rows) as candidate targets of CIC transcriptional regulation overlap with previously identified candidate targets (columns) in biologically distinct contexts. Genes in bold are found in more than one condition (row or column).

To gain insights into the biological role of CIC loss and its associated dysregulated gene expression patterns, we performed functional enrichment analyses. Biological processes significantly enriched for DE genes, classified into clusters of terms defined by similar gene sets, were dominated by those related to central nervous system (CNS) development and regulation (9/40, supplementary material, Table S5A). While this reflects CIC's role in nervous system development 16, 17, several clusters were also related to the development of other organs and systems, including the kidney, mammary gland, female reproductive system, and bone and vasculature. Given that CIC has been implicated in the development of several organ sites in *Drosophila* 13, 37, 38, 39 and mice 17, 40, 41, these results indicate that CIC may also play a more widespread and extensive role in human development than currently appreciated. Terms related to cell migration, chemotaxis, extracellular matrix organization, and cell adhesion may provide further insights into the mechanism by which CIC loss contributes to increased metastatic potential in lung cancer cells 35. Notably, several gene families had multiple members represented in these terms, such as protocadherin (*PCDH*) genes (which were universally underexpressed in *CIC* ^KO^ lines), and semaphorin, collagen, and annexin genes. Hallmark gene sets and oncogenic signatures significantly enriched for genes overexpressed in *CIC* ^KO^ lines included gene sets whose expression was found to increase upon activation of epidermal growth factor receptor (EGFR), ERBB2, RAF, KRAS, MEK, or mammalian target of rapamycin (MTOR) (supplementary material, Table S5B), implicating CIC in the control of these signalling pathways. Similarly, signatures significantly enriched for genes underexpressed in *CIC* ^KO^ lines included gene sets whose expression was found to decrease upon activation of KRAS, MEK, or MTOR, and upon knockdown of *RB*, *E2F1*, or *P53* (supplementary material, Table S5C).

### Transcriptome analysis of type I LGGs identifies high‐confidence candidate targets of CIC transcriptional regulation

**Figure 2:**
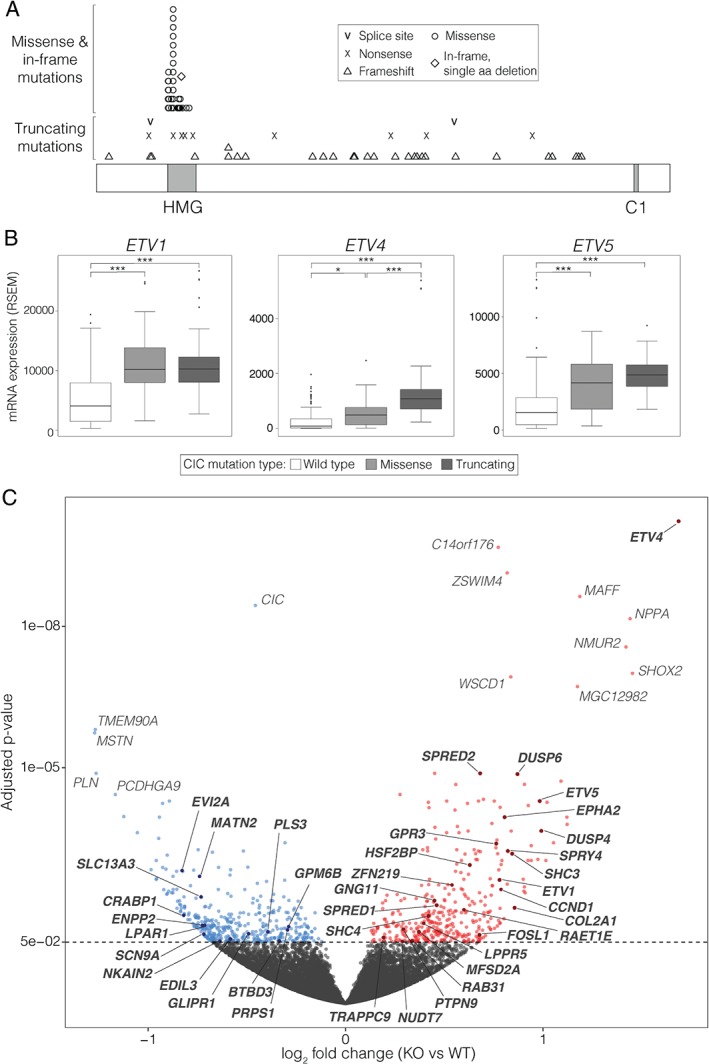
Transcriptome profiling identifies known and novel candidate targets of CIC transcriptional regulation. (A) Distribution of CIC mutations found in 78 type I LGG samples with CIC mutations from TCGA (supplementary material, Table S2). (B) Tukey boxplots showing gene expression for ETV1, ETV4 and ETV5 in type I LGGs from TCGA for samples with wild‐type CIC expression (n = 91), missense CIC mutations (n = 38), and truncating CIC mutations (n = 39). *p < 0.05 and ***p < 0.001 (two‐sided Student's t‐test) (C) Volcano plot of gene expression in type I LGGs with truncating CIC mutations (n = 39) compared to those with wild‐type CIC and high CIC expression (n = 68). High‐confidence candidate target genes (see Results) are labelled in bold (Table 2).

To explore the consequences of CIC deficiency in a primary tumour context, we obtained RNA‐sequencing data for type I LGGs from TCGA 42. Hemizygous *CIC* mutations found in type I LGGs show an interesting pattern, whereby ∼50% are truncating mutations distributed throughout the gene, and the remainder are missense mutations that cluster within the conserved HMG domain (Figure 2A) 42. To assess whether this distribution could be correlated with different patterns of transcriptional dysregulation, we analysed the expression of known CIC targets within tumour samples with missense (*CIC* ^mis^) or truncating (*CIC* ^trunc^) mutations. As expected, the expression of *ETV1*/*4*/*5* was significantly higher in *CIC* mutant samples than in *CIC* ^WT^ samples, regardless of mutation type (Figure 2B). However, *ETV4* also showed significantly higher expression in *CIC* ^trunc^ than in *CIC* ^mis^ samples, and a similar trend was observed for *ETV5*, indicating that CIC missense mutants may retain some repressive activity. To explore this possibility, we transfected *CIC* ^KO^ cells with FLAG‐tagged *CIC* constructs together with a luciferase reporter designed to drive expression through the *ETV5* promoter sequence (supplementary material, Figure S3). Luciferase activity following reintroduction of *CIC* constructs with missense mutations was reduced similarly to luciferase activity following reintroduction of *CIC* ^WT^, confirming that the mutant constructs retain some repressive activity. Conversely, reintroduction of a truncated form of *CIC* did not affect luciferase activity, consistent with complete loss of CIC's repressive activity.

**Table 2:**
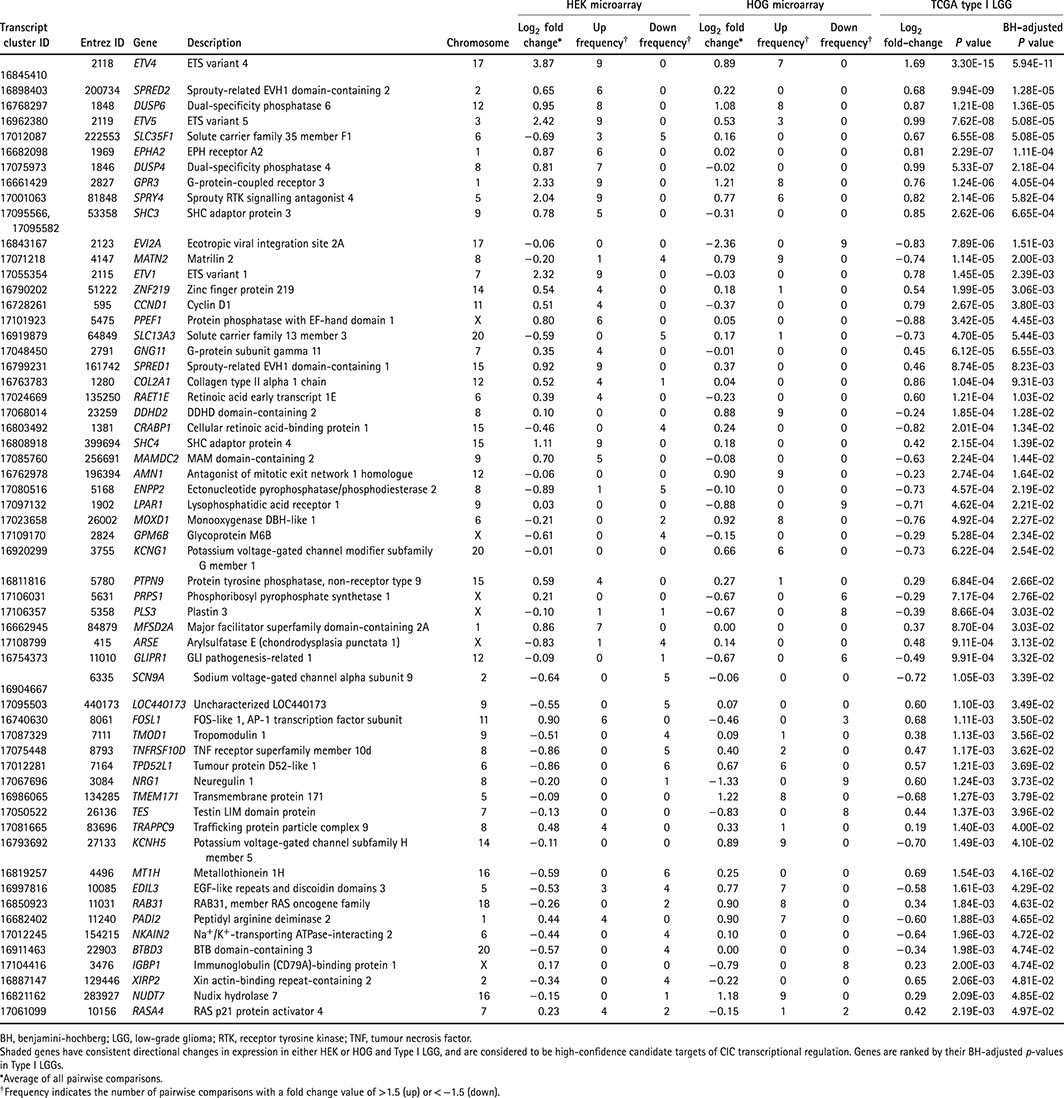
High‐confidence candidate targets of CIC transcriptional regulation

We therefore studied CIC's transcriptional network within the context of LGGs, comparing *CIC* ^trunc^ (*n* = 39) with *CIC* ^WT^ (*n* = 68) samples. A differential expression analysis identified 799 DE genes (FDR of < 5%; Figure 2C; supplementary material, Table S6). Although a similar analysis was performed previously 34, ours considered 84 additional samples and updated mutational annotations, in which the status of eight samples changed from *CIC* mutant to *CIC* ^WT^. Furthermore, whereas this earlier study exclusively reported genes showing increased expression in *CIC* mutant samples, our DE genes were approximately equally distributed between genes showing overexpression and underexpression in *CIC* ^trunc^ samples [380/799 (48%) and 419/799 (52%), respectively], which is consistent with the results obtained in our cell line models.

To identify genes whose differential expression was consistently associated with CIC loss, we analysed the overlap between DE genes obtained from our *CIC* ^KO^ lines and from type I LGGs (Table 2). Of the 58 genes that showed differential expression in primary tumour samples and in at least one cell line model, 39 (67%) had consistent directional changes (shaded in Table 2; Figure 2C). These 39 genes included the known CIC target genes *ETV1*, *ETV4*, and *ETV5*, along with 14 other genes previously reported to be candidate CIC targets (Table 1), and were considered to be high‐confidence candidate targets of CIC transcriptional regulation. It is of note that *ETV4*, *DUSP6*, *SPRY4* and *GPR3* showed increased expression in all three contexts. Importantly, the 19 genes that did not show consistent directional changes in expression may still represent direct or indirect targets of CIC, as CIC's transcriptional regulation activity may be, at least in part, context‐dependent. CIC's possible context dependency is further supported by the absence of an increase in the known targets *ETV1* and *ETV5* seen only in the HOG *CIC* ^KO^ lines.

### High‐confidence candidate targets show evidence of CIC regulation in isogenic cell line models

**Figure 3:**
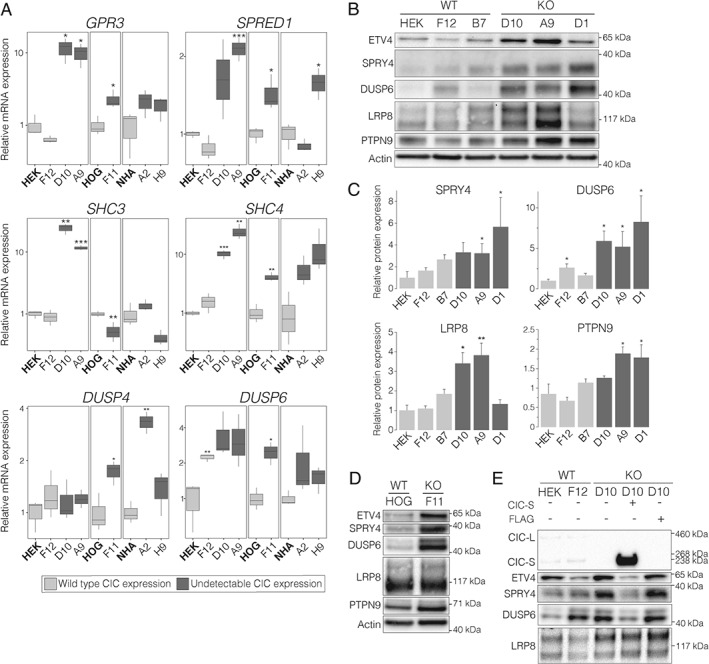
High‐confidence candidate targets of CIC regulation show increased transcript and protein expression in CIC ^KO^ cells. (A) Tukey boxplots showing expression of candidate target genes, as measured by RT‐qPCR, in the indicated cell lines compared to their respective parental cell lines (in bold). (B) Representative western blots showing increased expression of candidate CIC target genes in HEK‐derived CIC ^KO^ lines compared to CIC ^WT^ lines. Actin was used as a loading control, and a representative blot is shown. (C) Quantification of western blots for candidate CIC targets, showing mean relative expression compared to HEK cells. Additional quantifications are shown in supplementary material, Figure S4. All quantifications in (A) and (C) were obtained from three independent experiments. Error bars (C): standard error of the mean. *p < 0.05, **p < 0.01, and ***p < 0.001 (two‐sided Student's t‐test). (D) Representative western blots showing increased expression of candidate CIC target genes in the HOG‐derived CIC ^KO^ cell line compared to the parental cell line. (E) Representative western blots showing decreased expression of ETV4, SPRY4, and DUSP6 in a CIC ^KO^ cell line following reintroduction of CIC. A FLAG construct lacking CIC was used as a control.

To confirm the expression changes described above, a subset of the high‐confidence candidate targets were further validated at the mRNA and protein levels in the HEK‐derived and HOG‐derived *CIC* ^KO^ lines, along with additional *CIC* ^KO^ lines derived from a normal human astrocyte (NHA) line stably expressing wild‐type *IDH1* 43 (Figure 1C). mRNA levels for *GPR3*, *SPRED1*, *SHC3*, and *SHC4* were significantly increased in HEK‐derived *CIC* ^KO^ lines, and *DUSP4* and *DUSP6* showed similar trends (Figure 3A). *GPR3*, *SPRED1*, *SHC4*, *DUSP4*, and *DUSP6* also had significantly increased expression in the HOG‐derived *CIC* ^KO^ line, and all genes tested showed similar trends in the NHA‐derived *CIC* ^KO^ lines, reaching significance for *SPRED1* and *DUSP4*. Gene expression results were also confirmed by western blots, with SPRY4, LRP8, DUSP6, and PTPN9 showing significantly increased expression in HEK‐derived *CIC* ^KO^ lines (Figure 3B and supplementary materials, Figure S4), and ETV4, SPRY4 and DUSP6 showing increased expression in the HOG‐derived *CIC* ^KO^ line (Figure 3C).

To confirm that the increased protein expression of candidate targets is attributable to loss of CIC, we reintroduced *CIC* into one of the *CIC* ^KO^ lines. ETV4, SPRY4, and DUSP6 showed reduced expression upon reintroduction of *CIC*, but not upon introduction of an empty FLAG construct (Figure 3D), indicating that reintroduction of *CIC* is sufficient to suppress their expression. Interestingly, LRP8 expression remained similar upon reintroduction of *CIC*; given that CIC can function with a co‐repressor in *Drosophila* 11, 44, it is conceivable that a similar interaction occurs in humans, possibly also in a context‐dependent manner, and that this may be needed for effective repression of some of CIC's target genes. These results indicate that loss of CIC has potentially oncogenic functional consequences beyond transcriptional expression changes.

### Promoter regions associated with candidate target genes show evidence of CIC binding

**Figure 4:**
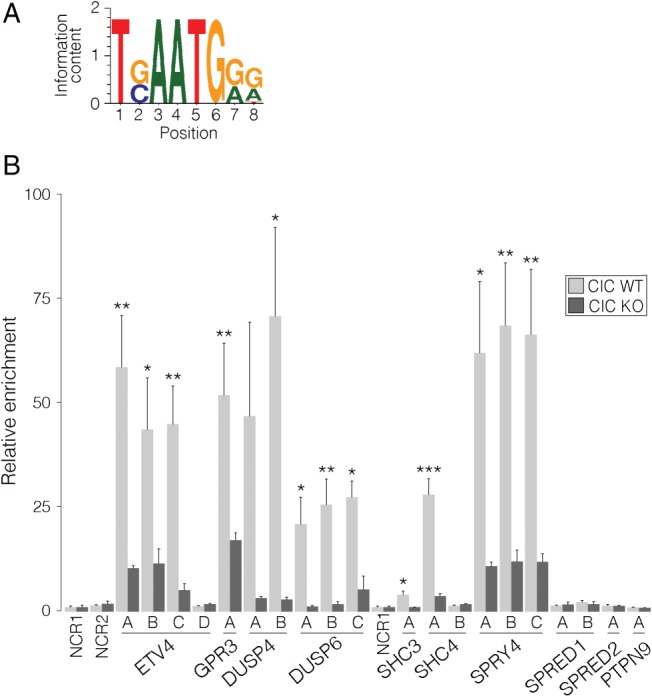
Promoter regions of high‐confidence candidate targets of CIC regulation show enrichment of CIC binding. (A) Consensus CIC binding sequence logo 11. (B) Mean enrichment of putative CIC binding sites relative to NCR1 following ChIP‐qPCR for CIC in CIC ^WT^ (HEK) and CIC ^KO^ (D10) cell lines. More detailed information can be found in supplementary material, Figure S5. Error bars: standard error of the mean over four (CIC ^WT^) or three (CIC ^KO^) independent experiments. qPCR analyses for each replicate had to be performed on two plates, and respective NCR1 values are shown. *p < 0.05, **p < 0.01, and ***p < 0.001 (two‐sided Student's t‐test).

To gauge whether the candidate CIC targets identified by our analyses were likely to be direct targets, we analysed their promoter regions [defined as 1500 bp upstream and 500 bp downstream of the transcription start site (TSS)] 45 to identify putative CIC binding sites. To do this, we made use of a previously defined CIC octameric consensus binding site (TG/CAATGG/AG/A; Figure 4A) 46. We performed our analyses allowing for one mismatch at position 2, 7, or 8 (i.e. the positions where sequence frequency is <100%). Genes identified as being DE in the HOG‐derived *CIC* ^KO^ lines (611 genes) or in *CIC* ^trunc^ type I LGGs (799 genes) were found to harbour significantly more of these putative binding sites in their promoters than genes showing no differences in expression (Fisher's exact test: *p* = 0.043 and *p* = 5.44 × 10^−5^, respectively). The 929 genes identified as being DE in the HEK‐derived *CIC* ^KO^ lines showed a similar trend (*p* = 0.090). Notably, high‐confidence candidate target genes were also associated with promoter regions that were significantly enriched for these putative binding sites (*p* = 0.036), indicating that they are likely to be enriched for direct targets. This notion is further supported by the presence within this list of CIC's known direct targets (*ETV1*, *ETV4*, and *ETV5*), whose promoters contain seven to 15 of these putative binding sites (Table 3).

**Table 3:** Number of putative CIC binding sites identified in the promoter regions of high‐confidence candidate target genes.

Gene	Entrez ID	No. of putative binding sites
MATN2	4147	19
ETV4	2118	15
SLC13A3	64849	14
SPRED2	200734	13
TPD52L1	7164	12
PLS3	5358	9
PTPN9	5780	8
ZNF219	51222	8
ETV1	2115	8
LPAR1	1902	8
DUSP6	1848	7
SHC4	399694	7
ETV5	2119	7
SCN9A	6335	6
ENPP2	5168	6
NKAIN2	154215	6
SPRY4	81848	5
BTBD3	22903	5
EPHA2	1969	4
GLIPR1	11010	4
PRPS1	5631	4
SHC3	53358	4
GPM6B	2824	3
DUSP4	1846	3
SPRED1	161742	3
EDIL3	10085	2
FOSL1	8061	2
RAB31	11031	1
COL2A1	1280	1
CCND1	595	1
NUDT7	283927	1
GPR3	2827	1
GNG11	2791	1
EVI2A	2123	1
PLPPR5	163404	1
CRABP1	1381	1
RAET1E	135250	1
MFSD2A	84879	0
TRAPPC9	83696	0

To confirm CIC binding in the promoter region of a subset of the high‐confidence candidate target genes, we performed targeted chromatin immunoprecipitation (ChIP) followed by quantitative polymerase chain reaction (qPCR) analysis. Putative CIC binding sites in the promoter regions of *ETV4*, *GPR3*, *DUSP4*, *DUSP6*, *SHC3*, *SHC4*, *SPRY4*, and *SPRED1* showed significant enrichment (∼2.5–80‐fold differences, *p* < 0.05) as compared with a negative control region (NCR) (NCR1; Figure 4B). Interestingly, three of the four sites tested in the *ETV4* promoter region showed significant enrichment (∼40–60‐fold differences), including one site in the promoter region of the shorter *ETV4* isoform (site D, uc002idv.5; supplementary material, Figure S5B). However, a second site in this same region (site C) did not show any enrichment, despite containing the same consensus sequence (TGAATGAA) as sites A and B. Of the other sites that did not show evidence of CIC binding, only half (3/6) had a single‐base mismatch to the CIC consensus sequence (*PTPN9* site A, TGAATGAT; *SHC4* site A, TAAATGGA; and *SPRED2* site A, TGAATGTG). However, two sites with a mismatch (*DUSP6* site C and *SHC3* site A, TTAATGAG) did show significant enrichment, suggesting that CIC can still bind in the presence of a mismatch, and may particularly tolerate a T at position 2. Importantly, CIC binding affinity may be further influenced by contextual elements such as the surrounding sequence, distance to the TSS, or cofactor binding; however, further genome‐wide studies will be needed to investigate these possibilities.

### CIC deficiency in biologically distinct contexts leads to dysregulation of similar pathways

*CIC* aberrations have recently begun to be associated with additional cancer types, such as sarcomas 19, 47, prostate cancer 36, and lung cancer 35. *CIC* is also significantly mutated in microsatellite instability (MSI) subtype STADs 48, and decreased CIC expression was found to correlate with disease stage in STAD samples, while overexpression of wild‐type CIC in a *CIC* ^mis^ STAD cell line decreased its invasive potential 35. To further characterize CIC's transcriptional network within distinct contexts and to investigate whether similar genes were affected by CIC deficiency in different cancer types, we identified genes whose differential expression was associated with loss of CIC in STAD 48. This yielded 1924 DE genes, including *ETV4* (FDR of <5% and FC of >1.5; supplementary material, Tables S7 and S8).

**Figure 5:**
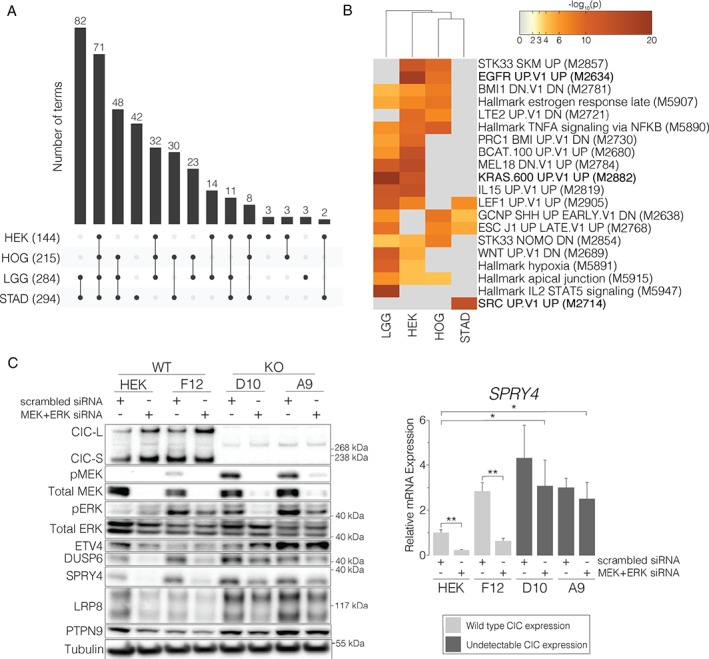
Gene expression differences associated with loss of CIC overlap with those associated with activation of MAPK signalling. (A) UpSet plot showing overlap of GO Biological Process terms significantly enriched for DE genes identified in the four contexts studied (Table S9A). Numbers in parentheses on the x‐axis indicate the number of terms enriched for DE genes identified in each context, and numbers above bar plots indicate the number of terms in each overlap displayed below. (B) The most enriched terms from the top 10 clusters of Hallmark gene sets and Oncogenic signatures enriched for genes that show overexpression upon loss of CIC (Table S9B). Term IDs from MSigDB are shown. Terms related to MAPK signalling are in bold. (C) Left: representative western blots of CIC ^WT^ and CIC ^KO^ cell lines treated with a ‘scrambled’ non‐targeting control siRNA or MEK‐specific and ERK‐specific siRNAs. Tubulin was used as a loading control, and a representative blot is shown. Right: quantification for SPRY4, shown as mean expression relative to HEK + scr siRNA. Additional quantifications are shown in supplementary material, Figure S6B. Error bars: standard error of the mean over three independent experiments. *p < 0.05, **p < 0.01, and ***p < 0.001 (two‐sided Student's t‐test).

To determine whether similar processes might be affected by CIC loss in different contexts, we performed a multi‐gene list functional enrichment analysis of genes identified as being DE in our cell line models and in primary samples (Figure 5A). Functional terms enriched for DE genes were similar to those seen in the cell line models (supplementary material, Table S5A), with a smaller proportion of clusters (5/40 versus 9/40) relating to CNS development (supplementary material, Table S9A). Notably, however, the majority of these CNS development‐related terms were significantly enriched in all four contexts, including *CIC*‐deficient STAD samples. Once again, clusters of terms related to the development of other organs and systems were present (i.e. vasculature and heart, muscle, bone, and female sexual development). Interestingly, terms related to the epithelial–mesenchymal transition (EMT) and the cellular response to hypoxia, both of which have been associated with invasiveness and treatment resistance in glioma 49, 50, 51, were also significantly enriched, along with additional terms related to mesenchymal development and angiogenesis. Disruptions in WNT–β‐catenin signalling and EMT also complement the apparent increase in cell motility conferred by loss of CIC 35. Genes overexpressed in *CIC*‐deficient samples showed enrichment of oncogenic signatures including gene sets that have been shown to be overexpressed upon activation of KRAS, EGFR, MEK, RAF, ERBB2, SRC, STK33, and CCND1 (Figure 5B; supplementary material, Table S9B). Hallmark gene sets related to upregulated KRAS signalling, hypoxia, and the p53 pathway were also significantly enriched. Consistent with these results, genes with reduced expression in *CIC*‐deficient samples were enriched for genes that have been shown to have reduced expression upon activation of KRAS, RAF, MEK, or CCND1, or upon downregulation of RB (supplementary material, Table S9C).

Taken together, these results indicate that the gene expression differences seen in *CIC*‐deficient samples are representative of gene expression dysregulation events frequently seen in various malignancies. They also show that, although the transcriptional consequences of CIC loss are, to some degree, context‐dependent (supplementary material, Figure S6A), the functional consequences of CIC loss appear to be similar across biologically distinct contexts (Figure 5A). This is consistent with the notion that *CIC* mutations play an oncogenic role and can do so beyond the context of LGG.

### Loss of CIC is associated with a MEK activation transcriptional signature

As noted, analyses of differential gene expression in *CIC*‐deficient cell line models and primary samples indicated that loss of CIC is associated with dysregulation of the MAPK signalling cascade. Indeed, several of the high‐confidence candidate target genes (*ETV1*/*4*/*5*, *DUSP4*/*6*, *SPRY4*, *SPRED2*, *GPR3*, *PTPN9*, and *LRP8*) have previously been identified as members of MEK 52, 53 and/or RAS 54 activation signatures. This may indicate that the transcriptional dysregulation associated with CIC loss overlaps with activation of the MAPK signalling cascade.

To test this hypothesis, we used small interfering RNAs (siRNAs) to knock down *MEK1*/*2* (*MEK*) and *ERK2* (*ERK*) expression in *CIC* ^WT^ and *CIC* ^KO^ lines. The expression of candidate CIC target genes (*SPRY4*, *DUSP6*, *LRP8*, and *PTPN9*) was reduced in *CIC* ^WT^ lines following *MEK*/*ERK* knockdown (Figure 5C; supplementary material, Figure S6B), consistent with results from previous studies 52, 53, 54. These results are also consistent with studies showing that ERK activity leads to CIC inhibition 11, 18; here, reduction of ERK activity could lead to relief of CIC inhibition, and thus to transcriptional repression of CIC target genes. Conversely, the expression of these target genes in *CIC* ^KO^ lines following *MEK*/*ERK* siRNA treatment is decreased to a lesser extent, indicating that active CIC is at least partially required to transduce changes in MEK/ERK activity. Furthermore, *MEK*/*ERK* siRNA treatment is generally unable to ‘rescue’ the expression of candidate target genes. Similar results were obtained following treatment with a MEK inhibitor (supplementary material, Figure S6C). Thus, loss of CIC leads to aberrant overexpression of downstream MAPK targets in the absence of other common MAPK‐activating mutations, indicating that it may present a novel mechanism for dysregulation of this common oncogenic pathway.

## Discussion

Here, we explored CIC's transcriptional network in novel isogenic cell line models and in two biologically distinct cancer types. We identified 39 high‐confidence candidate targets of CIC transcriptional regulation, including the established targets *ETV1*, *ETV4*, and *ETV5* 10, 11, 21, 34. We showed that this set of 39 genes appeared to be enriched for direct targets of CIC transcriptional regulation, and CIC binding in the promoter region of seven genes was confirmed by targeted ChIP‐qPCR analysis. Interestingly, our results indicate that CIC missense mutants may retain some repressive activity. While this study was focused on truncating *CIC* mutations within type I LGGs, further analyses exploring the transcriptional programmes associated with *CIC* missense mutations may further inform on the potential role of this class of mutations.

This study is also the first to report an extensive list of candidate targets of CIC transcriptional regulation in STADs. A comparison of DE genes identified in biologically distinct contexts revealed that, although only *ETV4* was common to all contexts studied, similar biological processes and gene families appeared to be consistently affected. For instance, we observed several members of the *PCDH* gene family showing decreased expression in *CIC*‐deficient samples. Reduced expression of several *PCDH* genes has been implicated in both low‐grade and high‐grade gliomas, including *PCDHGA11* 55, *PCDH10* 56, and *PCDH9* 57, 58, 59. Similarly, hypermethylation and associated decreased expression of *PCDH10* 60, 61, 62, *PCDH8* 63 and *PDCH17* 64, 65 have been associated with poor prognosis in gastric cancers. Thus, loss of CIC may affect cell adhesion processes through gene expression dysregulation, which is consistent with a recent report showing that loss of CIC in lung cancer cells leads to increased metastatic potential through elevated expression of ETV4 and matrix metalloproteinase‐24 (MMP24) 35. Other common pathways included the development of several tissue types, indicating that CIC may be more extensively involved in human development than currently appreciated.

We also observed an enrichment of known RTK–MAPK pathway regulators within DE genes, consistent with the notion that CIC may function in one or more feedback loop(s) to regulate MAPK signalling, as previously suggested 10, 34. Functional enrichment analyses also indicated that gene expression changes that occur upon loss of CIC significantly overlap with those that occur in response to increased MAPK signalling. We showed that MEK/ERK inhibition was able to reduce the expression of targets in *CIC* ^WT^ lines, but less so or not at all in *CIC* ^KO^ lines, indicating that CIC is needed, at least in part, to transduce signals from upstream members of the MAPK signalling pathway. Our results, combined with the observation that *CIC* mutations rarely co‐occur with other activating alterations in this pathway 8, indicate that loss of CIC may provide a novel mechanism for activation of downstream members of the MAPK signalling cascade. These results are consistent with recent reports showing that loss of CIC imparts resistance to MAPK and EGFR inhibitors in various cancer‐derived cell lines with activating mutations in upstream members of the pathway, including KRAS, NRAS, BRAF, and EGFR 23, 24. Although these reports show that increased expression of *ETV1*/*4*/*5* contributes to this phenotype, the additional CIC targets identified in our study may also play a role in this response. Our results thus expand on the potential roles of *CIC* mutations in malignancy, and may provide new insights into the possible mechanisms underlying phenotypic responses recently associated with CIC loss, such as shorter times to recurrence, increased metastatic potential, and resistance to MAPK inhibitors 9, 10, 23, 24, 35.

## Author contributions statement

VGL, MF, SC, MAM: conceived and designed the study; VGL: performed bioinformatics analyses and, along with MAM, wrote the manuscript; MF: developed the ZFN *CIC* knockout cell line; JS, SYC, AL, SC: developed the CRISPR/Cas9 *CIC* knockout cell lines; JS, SYC: performed most cell line‐based experiments; MAM: supervised the project; SC, SY: provided further guidance. All authors participated in discussions regarding the experiments and results, and reviewed and approved the manuscript.

**SUPPLEMENTARY MATERIAL ONLINE**

**Supplementary materials and methods**

**Supplementary figure legends**

**Figure S1.** *CIC* expression in Type I LGGs with intact *CIC* (WT) or truncating *CIC* mutations (Mut)

**Figure S2.** Generation of *CIC* knockout cell lines

**Figure S3.** *CIC* missense mutants retain repressive activity

**Figure S4.** ETV4 shows increased protein expression in *CIC* ^KO^ cell lines

**Figure S5.** Targeted ChIP‐qPCR analysis of high‐confidence candidate targets of CIC

**Figure S6.** CIC loss leads to increased expression of downstream MAPK targets

**Table S1.** Antibody and primer information

**Table S2.** TCGA samples used for analyses

**Table S3.** Differential expression analysis results from HEK‐derived *CIC* knockout cell lines

**Table S4.** Differential expression analysis results from the HOG‐derived *CIC* knockout cell line

**Table S5.** Functional enrichment results for genes differentially expressed in *CIC* knockout cell lines compared to *CIC* wild type cell lines

**Table S6.** Differential expression analysis results for Type I LGGs

**Table S7.** Differential expression analysis results for STAD samples

**Table S8.** Overlap of differentially expressed genes

**Table S9.** Functional enrichment results for genes differentially expressed in *CIC*‐deficient samples

## Supporting information


**Supplementary materials and methods**

**Supplementary figure legends**

**Figure S1. Generation of CIC knockout cell lines**. (**A**) Scheme illustrating the generation of CIC knockout cell lines using the ZFN and CRISPR/Cas9 systems. (**B**) Protein structure of the CIC isoforms (short [CIC‐S] and long [CIC‐L]) annotated with conserved domains. N1: conserved N‐terminal domain. HMG: DNA‐binding high mobility group box domain. C1: conserved C‐terminal domain. (**C**) Additional Western blot showing lack of CIC expression in CIC knockout cell lines (see Figure 1A).

**Figure S2. CIC expression in Type I LGGs with intact CIC (WT) or truncating CIC mutations (Mut)**. Dotted line indicates the 1^st^ quartile expression cutoff for WT samples.

**Figure S3. CIC missense mutants retain repressive activity**. (**A**) Representative Western blot of cells used for the luciferase assays. D1 cells were transfected with the indicated constructs, and TBP was used as a loading control. R201W and R1515H are missense mutations in the HMG and C1 domains, respectively. Q564X is a nonsense mutation that results in a truncated form of CIC. (**B**) Diagram of the relevant portion of the luciferase reporter construct used. The numbers represent distance (in bp) from the ETV5 transcription start site. (**C**) Relative luciferase expression in cells transfected with indicated CIC‐S constructs. Loss of CIC‐mediated repression is clear in the Q564X nonsense mutation, while the missense mutants retain repressive activity similar to the wild type construct. Error bars: s.e.m. over three independent experiments. *p < 0.05, **p < 0.01, ***p < 0.001 compared to the vector‐only control (two‐sided Student's t‐test).

**Figure S4. ETV4 shows increased protein expression in CIC^KO^ cell lines**. Quantification for Western blot shown in Figure 3B. Error bars: s.e.m. over three independent experiments.

**Figure S5. Targeted ChIP‐qPCR analysis of high‐confidence candidate targets of CIC**. Zoomed‐in views of Figure 4B for each putative CIC binding site tested. Isoforms were obtained from the UCSC genome browser (Hg38), and respective IDs are shown. Chromosomal locations are also indicated. The sequence found within each site is indicated, with mismatches underlined. Bar plots show relative enrichment of each site compared to NCR1 in CIC ^WT^ samples (light grey) and CIC ^KO^ samples (dark grey). NCR1 and NCR2 (not shown) are located ∼1 kb upstream of ETV4 Site A and ∼1 kb downstream of ETV4 Site C, respectively. Red and blue bars indicate sites found on the positive and negative strands, respectively. Error bars: s.d. over four (WT) or three (KO) independent experiments. *p < 0.05, **p < 0.01, ***p < 0.001

**Figure S6. CIC loss leads to increased expression of downstream MAPK targets**. (**A**) UpSet plot showing overlap of DE genes in the four contexts we studied. (**B**) Additional quantifications for Western blots shown in Figure 5C, shown relative to HEK + scr siRNA . Error bars: s.e.m. over three independent experiments. *p < 0.05, **p < 0.01 (two‐sided Student's t‐test). (**C**) Representative Western blots of indicated cell lines treated with a vehicle control (DMSO) or a MEK inhibitor (Trametinib). Results from this treatment are consistent with results seen following MEK/ERK knockdown using siRNAs (Figure 5C). Tubulin was used as a loading control, and a representative blot is shown.

**Table S1. TCGA samples used for analyses**.

**Table S2. Differential expression analysis results from HEK‐derived CIC knockout cell lines**. Frequency indicates the number of pairwise comparisons in which the gene showed an absolute fold change value > 1.5. The log^2^ fold change value showed is the average of all nine comparisons.

**Table S3. Differential expression analysis results from the HOG‐derived CIC knockout cell line**. Frequency indicates the number of pairwise comparisons in which the gene showed an absolute fold change value > 1.5. The log_2_ fold change value showed is the average of all nine comparisons.

**Table S4. Functional enrichment results for genes differentially expressed in CIC knockout cell lines compared to CIC wild type cell lines**. (**a**) All DE genes, testing enrichment for Gene Ontology (GO) biological processes. (**b**) Genes whose expression increases in CIC knockout cell lines, testing enrichment for Hallmark gene sets and Oncogenic signatures. (**b**) Genes whose expression decreases in CIC knockout cell lines, testing enrichment for Hallmark gene sets and Oncogenic signatures.

**Table S5. Differential expression analysis results for Type I LGGs**. Results from the DESeq2 analysis performed between samples with truncating CIC mutations and samples with intact CIC and high CIC expression.

**Table S6. Differential expression analysis results for STAD samples**. Results from the DESeq2 analysis performed between samples with CIC copy number loss and samples with neutral CIC copy number.

**Table S7. Overlap of differentially expressed genes**. (**a**) Genes identified as DE in the CIC knockout cell lines and in STAD samples. (**b**) Genes identified as DE in Type I LGGs and in MSI subtype STAD samples. Shaded genes have consistent directional changes in expression.

**Table S8. Functional enrichment results for genes differentially expressed in CIC‐deficient samples**. (**a**) All DE genes, testing enrichment for Gene Ontology (GO) biological processes. (**b**) Genes whose expression increases in CIC‐deficient samples, testing enrichment for Hallmark gene sets and Oncogenic signatures. (**b**) Genes whose expression decreases in CIC‐deficient samples, testing enrichment for Hallmark gene sets and Oncogenic signatures.

**Table S9. Antibody and primer information**. (a) Antibody information. (b) RT‐qPCR and ChIP‐qPCR primer information.
