# Supplementary material for: Comparative transcriptome analysis of isogenic cell line models and primary cancers links capicua (CIC) loss to activation of the MAPK signalling cascade
Source: J Pathol. 2017 Apr 26;242(2):206–20. doi: 10.1002/path.4894 (PMC5485162; doi:10.1002/path.4894)
Supplement: Supplementary file 1 — Supplementary materials and methods [file PATH-242-206-s001.pdf]

Materials and Methods

Cell culture and conditions

The HEK293a cell line was obtained from Dr Gregg Morin (Canada's Michael Smith Genome Sciences Centre, Vancouver, BC, Canada) and authenticated by Genetica DNA Laboratories (Cincinnati, OH, USA). The HOG cell line was obtained from Dr G. Dawson (The University of Chicago, Illinois, USA), and the IDH1-stable NHA cell line was obtained from Applied Biological Materials (ABM) Inc (T3022; Richmond, BC, Canada). The lines were shown to be free of *Mycoplasma* contamination using the e-Myco mycoplasma PCR detection kit (iNtRON). Unless otherwise stated, at ~70-90% confluency, cells were washed with PBS, trypsinized, and either passaged or harvested by pelleting.

Whole-cell lysate extraction

Cells were thawed on ice and resuspended in 3-5X packed-cell volume of ice-cold RIPA Lysis Buffer System (Santa Cruz Biotechnology, Dallas, TX, USA) according to the manufacturer's instructions and freshly supplemented with 1X PhosSTOP phosphatase inhibitor cocktail when applicable (Roche [Mississauga, ON, Canada]). Cell pellets were homogenized by 5-10 passages through a 21-gauge needle then mixed for 60 minutes at 4 °C on an automatic rotator. Insoluble cellular debris was pelleted using centrifugation at 13000 rpm for 10 mins at 4 °C.

Western blotting

Samples were subjected to gel electrophoresis on NuPage 3-8% Tris Acetate pre-cast mini-gels (CIC, LRP8, pERK, and total ERK; Life Technologies [Ottawa, ON]) or NuPage 10% BisTris pre-cast mini-gels (ETV4, SPRY4, DUSP6, PTPN9, pMEK, and total MEK; Life Technologies) with 1X MOPs buffer (Life Technologies) containing 1X reducing agent (Life Technologies) and antioxidant (Life Technologies) at 100V for 15 minutes, followed by 150V for 2-3 hours (CIC) or 1.5-2 hours (ETV4, SPRY4, DUSP6, PTPN9, LRP8, pMEK, total MEK, pERK, total ERK). Separated proteins were transferred onto a methanol-activated PVDF membrane (Pall [St Laurent, QC, Canada] or Bio-Rad [Mississauga, ON]) for 60-90 minutes at 100V in 1X transfer buffer (Life Technologies) with 20% (v/v) methanol. Membranes were blocked with 2% (w/v) skim milk in PBST with 0.1% Tween 20 for one hour at room temperature, followed by overnight incubation with primary antibodies at 4°C. For protein signal detection, membranes were incubated with secondary antibodies for one hour at room temperature, followed by application of either ECL substrate (Bio-Rad) or SuperSignal West Femto substrate (ThermoFisher Scientific [Ottawa, ON]). Antibody information can be found in Table S1A. Images were captured using a ChemiDoc^{TM} MP Imager (Bio-Rad), and protein quantifications were performed using Image Lab 4.1 software (Bio-Rad).

A CIC^{KO} *line constructed using the ZFN approach*

The D1 *CIC*^{KO} line used in this study was derived using two rounds of zinc finger nuclease (ZFN) treatment. HEK cells were co-transfected with the following: 5 µL of custom-designed CIC-specific CompoZr^{TM} custom ZFN construct mRNA (Sigma-

Aldrich [Oakville, ON]) with a target site of GCCTCCAACCAGAGCaaaggtGAGGGCTGGTGGGGACTG (with the two ZFN-binding half sites capitalized); and 250 ng of a Hygro RS reporter construct (Toolgen [Seoul, South Korea]) harbouring the same ZFN target site to enrich for cells in which ZFNs are active. Transfection was performed by seeding 4 x 10^5 cells in a 6-well plate ~24 hours prior to transfection and performing lipid-based transfection using the Trans-IT-mRNA transfection reagent and Trans-IT Boost reagent (Mirius Bio [Madison, WI, USA]) according to Sigma's recommendations for CompoZr™ custom ZFN transfection. Enrichment for cells with active ZFNs was carried out by treating cells with 0.5 mg/mL hygromycin at ~72-96 hours post-transfection. From the resulting enriched cell population, single clones were isolated using a limiting dilution method.

CIC^{WT} *and* CIC^{KO} *lines constructed using the CRISPR/Cas9 approach*

The following cell lines were derived using CRISPR/Cas9 treatment: B7 and F12 (*CIC*^{WT}) and A9 and D10 (*CIC*^{KO}) from HEK, F11 (*CIC*^{KO}) from HOG, and A2 and H9 (*CIC*^{KO}) from NHA. CRISPR guide sequences were designed against exon 2 and 4 of the *CIC* gene using the online CRISPR Design tool (http://crispr.mit.edu) provided by the Zhang laboratory. The guides were then cloned into CRISPR-Cas9 expression vector pSpCas9(BB)-2A-GFP (PX458) (Addgene [Cambridge, MA, USA]) following the Zhang protocol (http://www.genome-engineering.org/crispr/wp-content/uploads/2014/05/CRISPR-Reagent-Description-Rev20140509.pdf). The *CIC* target sequences used were as follows: CIC-S_exon2_DBSg1T (5'-CACCGCCCCTCCGTGCAGCCGAGCG-3'), CIC-S_exon2_DBSg1B (5'-

AAACCGCTCGGCTGCACGGAGGGGC -3'), CIC-S_exon4_DBSg1T (5'-CACCGCGACGTTTTCCGGGCGGTAG-3') and CIC-S_exon4_DBSg1B (5'-AAACCTACCGCCCGGAAAACGTCGC-3'). HEK293a cells were transfected with the vector containing sgRNA against *CIC* and cells expressing GFP protein were single-cell sorted using FACS into 96 well plates. After 2 weeks, colonies originating from single cells were identified and transferred to 6-well plates at 70% confluency.

For the ZFN and CRISPR/Cas9 lines, isolated clones were screened for CIC expression using Western blot analysis with an anti-CIC antibody (Table S1A). Clones without CIC-S and CIC-L protein expression were further Sanger sequenced to confirm genome editing (insertion or deletion) in the targeted area.

siRNA transfections

For *CIC* knockdown, HEK cells (1.5 x 10^5) were transfected with 50 nM *CIC* siRNA (HSS118258, ThermoFisher Scientific) and 5 µL Lipofectamine® RNAiMAX Transfection Reagent (Life Technologies). Cells were harvested 48 hours post-transfection. For *MEK*/*ERK* knockdown, cells (2.25 x 10^5 for HEK, 2.5 x 10^5 for F12, and 2.875 x 10^5 for D10 and A9) were transfected with 20 nM each of *MEK1* (VHS40795, ThermoFisher Scientific) and *MEK2* (VHS40800) siRNA or 40 nM non-specific negative control siRNA (12935300) and 7.5 µL Lipofectamine® RNAiMAX Transfection Reagent. At 48 hours post-transfection, the cells were further transfected with 10 nM *MEK1* and 20 nM *ERK2* (VHS40312) siRNA or control siRNA and harvested after a further 24 hours.

RT-qPCR

RNA extraction was performed using the RNeasy Plus Mini Kit (74136, Qiagen) according to the manufacturer's recommendations. RT-qPCR was carried out using 50 ng of template RNA with the *Power* SYBR® Green RNA-to-C_t^{TM}*1-Step* Kit (4389986, Life Technologies) according to the manufacturer's recommended reaction component amounts and cycling conditions. A 7900 HT Sequence Detection System with SDS 2.4 software was used for temperature cycling and the generation of C_T values. Analysis of relative mRNA expression was performed using the 2-$\Delta\Delta C_T$ method with TATA-box binding protein (*TBP*) expression as an endogenous control, and two-sided Student's *t*-tests were used to assess differential expression for experiments performed on lysates obtained from three separate passages. Sequences of the RT-qPCR primers used are provided in Table S1B.

FLAG-CIC construct generation and luciferase assays

CIC constructs were generated as previously described [32], with the following primers used for site-directed mutagenesis for the Q564X construct: 5'-CAGGAGTCTGGTTAGGGCAGCACAG-3' (forward) and 5'-CTGTGCTGCCCTAACCAGACTCCTG-3' (reverse). Re-introduction of constructs into *CIC*^{KO} lines was performed as previously described [33]. ~24 hrs prior to transfection, 1.4 x 10^5 cells per transfection were seeded in 24-well plates. Transfection was carried out using the Turbofect transfection reagent (R0531, ThermoFisher Scientific) according to the manufacturer's recommendations. The following DNA amounts were transfected into each well: 0.6 µg of a pGL3 reporter vector with a cloned promoter sequence of *ETV5*

(99 to 996 base pairs upstream of the transcription start site), 40 ng of the indicated flag-*CIC* construct or corresponding flag vector-only control, and 0.1 μg of a pRL-CMV vector (E2261, Promega) as a transfection control. At ~72 hours post-transfection, luciferase expression was measured using the Dual Glo luciferase assay system (E2920, Promega) according to the manufacturer's recommendations and with a Perkin Elmer Wallac 1420 Victor2 microplate reader.

Trametinib treatment

3 x 10^5 cells were seeded in a 6-well plate. The following day, media was replaced with either 0.045% DMSO or 1μM Trametinib in DMEM. After 24 hours, cells were harvested for Western blot analysis.

Promoter analysis

To identify putative CIC binding sites within the promoters of differentially expressed genes, we made use of the R/Bioconductor package GenomicFeatures [66] (version 1.22.7) to extract promoter sequences, defined as 1.5 kilobases (kb) upstream and 0.5 kb downstream of the transcription start site. We then used the R/Bioconductor package Biostrings (version 2.38.1) to identify instances of the *Drosophila* CIC consensus binding sequence [67]. A minimum score of 88% was used to allow for mismatches at sites with <100% position frequency. The isoform with the most CIC binding sites was retained for further analyses. Fisher's exact tests were used to assess enrichment within gene lists relative to the rest of the genome, comparing genes with at least one putative binding site in their promoter regions to genes with no sites [68].

Targeted ChIP-qPCR analysis

For each cell line and replicate, two ~70-80% confluent 15 cm plates were treated with 1% formaldehyde (Sigma) in PBS for 12 minutes with gentle rocking, followed by treatment with 0.125 M glycine (Sigma) for five minutes. Crosslinked cells were combined and pelleted using centrifugation at 1200 rpm for 5 minutes at room temperature, resuspended in 450 μL ChIP lysis buffer (50mM Tris-HCl pH 8.0, 1% SDS, 10mM EDTA, 1X cOmplete EDTA-free Protease Inhibitor cocktail [PIC, Roche]), and lysed on ice for 30 minutes. Cells were homogenized by 6 passages through a 20-gauge needle, and nuclei were pelleted by centrifugation at 5000 rpm for 10 minutes at 4°C. The pellet was resuspended in 900 μL shearing buffer (10mM Tris-HCl pH 8.0, 0.1% SDS, 1mM EDTA, 1X EDTA-free PIC) and transferred to a 1 mL milliTUBE with AFA fiber (D-Mark Biosciences) using a 30-gauge needle. Chromatin was sonicated using a Covaris S2 sonicator using the following settings: 10% duty cycle, 6 intensity, 500 burst, 16 cycles (20s on, 40s off) at 4-6°C. Insoluble debris and unfragmented chromatin were removed by centrifugation at 14000 rpm for 12 minutes at 4°C. An aliquot of chromatin was de-crosslinked overnight at 68°C (0.2 M NaCl, 0.05 mg/mL RNase), treated with proteinase K for 30 min at 42°C, and purified using the MinElute PCR Purification Kit (28006, Qiagen) to allow determination of chromatin concentration using the Qubit dsDNA high sensitivity assay (Life Technologies) and to confirm that DNA fragments were present in the 150-300 bp size range through separation on a 2% agarose gel. Protein A Dynabeads (Life Technologies) blocked with bovine serum albumin and salmon sperm DNA for 3 h were used to pre-clear chromatin for 2 h. For the

immunoprecipitation, three volumes of IP buffer (10 mM Tris-HCl pH 8.0, 1% Triton X-100, 0.1% deoxycholate, 0.1% SDS, 90 mM NaCl, 2mM EDTA, 1X EDTA-free PIC) were added to 22.5 μg of chromatin, which was then incubated with 5 μg of anti-CIC antibody (Table S1A) for 1h at 4°C. Blocked Protein A Dynabeads (25 μL) were then added to the chromatin and antibody and incubated overnight at 4°C. The samples were then washed twice in low salt buffer (20mM Tris-HCl pH 8.0, 0.1% SDS, 1% Triton X-100, 2 mM EDTA, 150 mM NaCl) and twice in high salt buffer (same as low salt buffer except 300 mM NaCl). DNA was eluted in 100 mM sodium bicarbonate with 1% SDS and 0.05 mg/mL RNase at 68°C for 6 hours or overnight, followed by treatment with proteinase K for 30 min at 42°C. For each IP, 20 μL of chromatin was subject to the same treatment to serve as input controls. Eluted DNA was purified using the MinElute PCR Purification Kit, and qPCR analysis was performed as described above using the *Power* SYBR® Green PCR Master Mix (4367659, Life Technologies) according to the manufacturer's recommended reaction component amounts and cycling conditions. Percent input values were calculated for each site, and reported as fold-change differences compared to NCR1. Two-sided Student's *t*-tests were used to assess differential expression for experiments performed on cells obtained from four (HEK) or three (D10) separate passages. Sequences of the qPCR primers used are provided in Table S1B.

Functional enrichment analysis

The Metascape software (http:/metascape.org) was used to perform functional enrichments using the multiple gene lists mode [69]. GO Biological Processes were used

for enrichment analyses of all DE genes, and Hallmark Gene Sets and Oncogenic Signatures were used for enrichment analyses of over- or under-expressed genes, with a minimum overlap of 3 genes, a p-value cutoff of 0.05, and a minimum enrichment of 1.5. Only terms with a BH-adjusted p-value < 0.05 were retained.
