# Supplementary material for: Comparative transcriptome analysis of isogenic cell line models and primary cancers links capicua (CIC) loss to activation of the MAPK signalling cascade
Source: J Pathol. 2017 Apr 26;242(2):206–20. doi: 10.1002/path.4894 (PMC5485162; doi:10.1002/path.4894)
Supplement: Supplementary file 2 — Supplementary figure legends [file PATH-242-206-s002.doc]

**Figure S1. Generation of *CIC* knockout cell lines.** (**A**) Scheme illustrating the generation of *CIC* knockout cell lines using the ZFN and CRISPR/Cas9 systems. (**B**) Protein structure of the CIC isoforms (short [CIC-S] and long [CIC-L]) annotated with conserved domains. N1: conserved N-terminal domain. HMG: DNA-binding high mobility group box domain. C1: conserved C-terminal domain. (**C**) Additional Western blot showing lack of CIC expression in *CIC* knockout cell lines (see Figure 1A).

**Figure S2. *CIC* expression in Type I LGGs with intact *CIC* (WT) or truncating *CIC* mutations (Mut).** Dotted line indicates the 1^st^ quartile expression cutoff for WT samples.

**Figure S3. *CIC* missense mutants retain repressive activity.** (**A**) Representative Western blot of cells used for the luciferase assays. D1 cells were transfected with the indicated constructs, and TBP was used as a loading control. R201W and R1515H are missense mutations in the HMG and C1 domains, respectively. Q564X is a nonsense mutation that results in a truncated form of CIC. (**B**) Diagram of the relevant portion of the luciferase reporter construct used. The numbers represent distance (in bp) from the *ETV5* transcription start site. (**C**) Relative luciferase expression in cells transfected with indicated CIC-S constructs. Loss of CIC-mediated repression is clear in the Q564X nonsense mutation, while the missense mutants retain repressive activity similar to the wild type construct. Error bars: s.e.m. over three independent experiments. *p < 0.05, **p < 0.01, ***p < 0.001 compared to the vector-only control (two-sided Student’s *t*-test).

**Figure S4. ETV4 shows increased protein expression in *CIC*^KO^ cell lines.** Quantification for Western blot shown in Figure 3B. Error bars: s.e.m. over three independent experiments.

**Figure S5. Targeted ChIP-qPCR analysis of high-confidence candidate targets of CIC.** Zoomed-in views of Figure 4B for each putative CIC binding site tested. Isoforms were obtained from the UCSC genome browser (Hg38), and respective IDs are shown. Chromosomal locations are also indicated. The sequence found within each site is indicated, with mismatches underlined. Bar plots show relative enrichment of each site compared to NCR1 in *CIC*^WT^ samples (light grey) and *CIC*^KO^ samples (dark grey). NCR1 and NCR2 (not shown) are located ~1kb upstream of *ETV4* Site A and ~1kb downstream of *ETV4* Site C, respectively. Red and blue bars indicate sites found on the positive and negative strands, respectively. Error bars: s.d. over four (WT) or three (KO) independent experiments. *p < 0.05, **p < 0.01, ***p < 0.001

**Figure S6. CIC loss leads to increased expression of downstream MAPK targets.** (**A**) UpSet plot showing overlap of DE genes in the four contexts we studied. (**B**) Additional quantifications for Western blots shown in Figure 5C, shown relative to HEK + scr siRNA . Error bars: s.e.m. over three independent experiments. *p < 0.05, **p < 0.01 (two-sided Student’s *t*-test). (**C**) Representative Western blots of indicated cell lines treated with a vehicle control (DMSO) or a MEK inhibitor (Trametinib). Results from this treatment are consistent with results seen following *MEK*/*ERK* knockdown using siRNAs (Figure 5C). Tubulin was used as a loading control, and a representative blot is shown.
