# Supplementary material for: Comparative transcriptome analysis of isogenic cell line models and primary cancers links capicua (CIC) loss to activation of the MAPK signalling cascade
Source: J Pathol. 2017 Apr 26;242(2):206–20. doi: 10.1002/path.4894 (PMC5485162; doi:10.1002/path.4894)
Supplement: Supplementary file 3 — Figure S1. Generation of CIC knockout cell lines. (A) Scheme illustrating the generation of CIC knockout cell lines using the ZFN and CRISPR/Cas9 systems. (B) Protein structure of the CIC isoforms (short [CIC‐S] and long [CIC‐L]) annotated with conserved domains. N1: conserved N‐terminal domain. HMG: DNA‐binding high mobility group box domain. C1: conserved C‐terminal domain. (C) Additional Western blot showing lack of CIC expression in CIC knockout cell lines (see Figure 1A). [file PATH-242-206-s003.pdf]

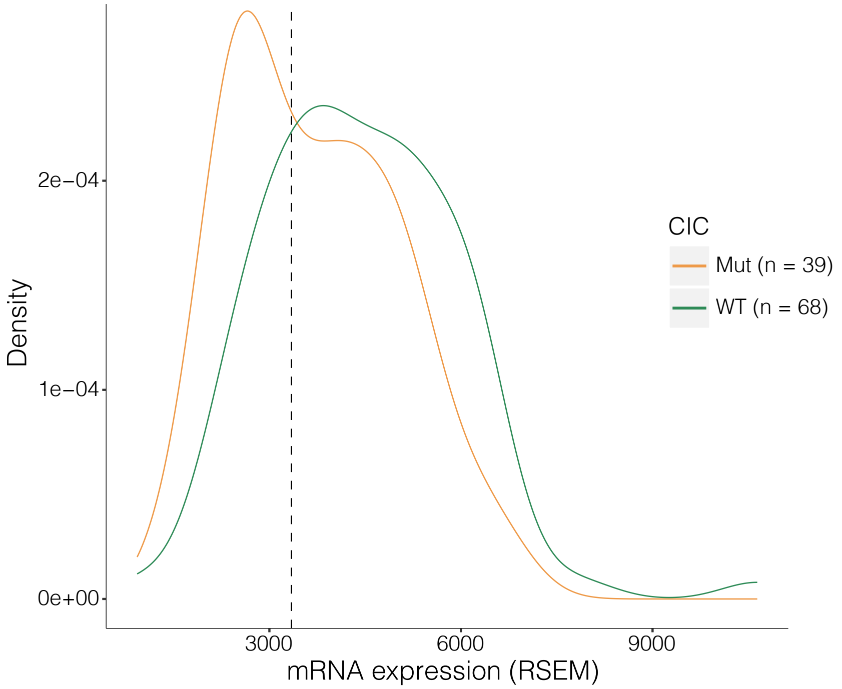

Figure S1. *CIC* expression in Type I LGGs with intact *CIC* (WT) or truncating *CIC* mutations (Mut). Dotted line indicates the 1st quartile expression cutoff for WT samples.
