# Supplementary material for: Comparative transcriptome analysis of isogenic cell line models and primary cancers links capicua (CIC) loss to activation of the MAPK signalling cascade
Source: J Pathol. 2017 Apr 26;242(2):206–20. doi: 10.1002/path.4894 (PMC5485162; doi:10.1002/path.4894)
Supplement: Supplementary file 4 — Figure S2. CIC expression in Type I LGGs with intact CIC (WT) or truncating CIC mutations (Mut). Dotted line indicates the 1st quartile expression cutoff for WT samples. [file PATH-242-206-s004.pdf]

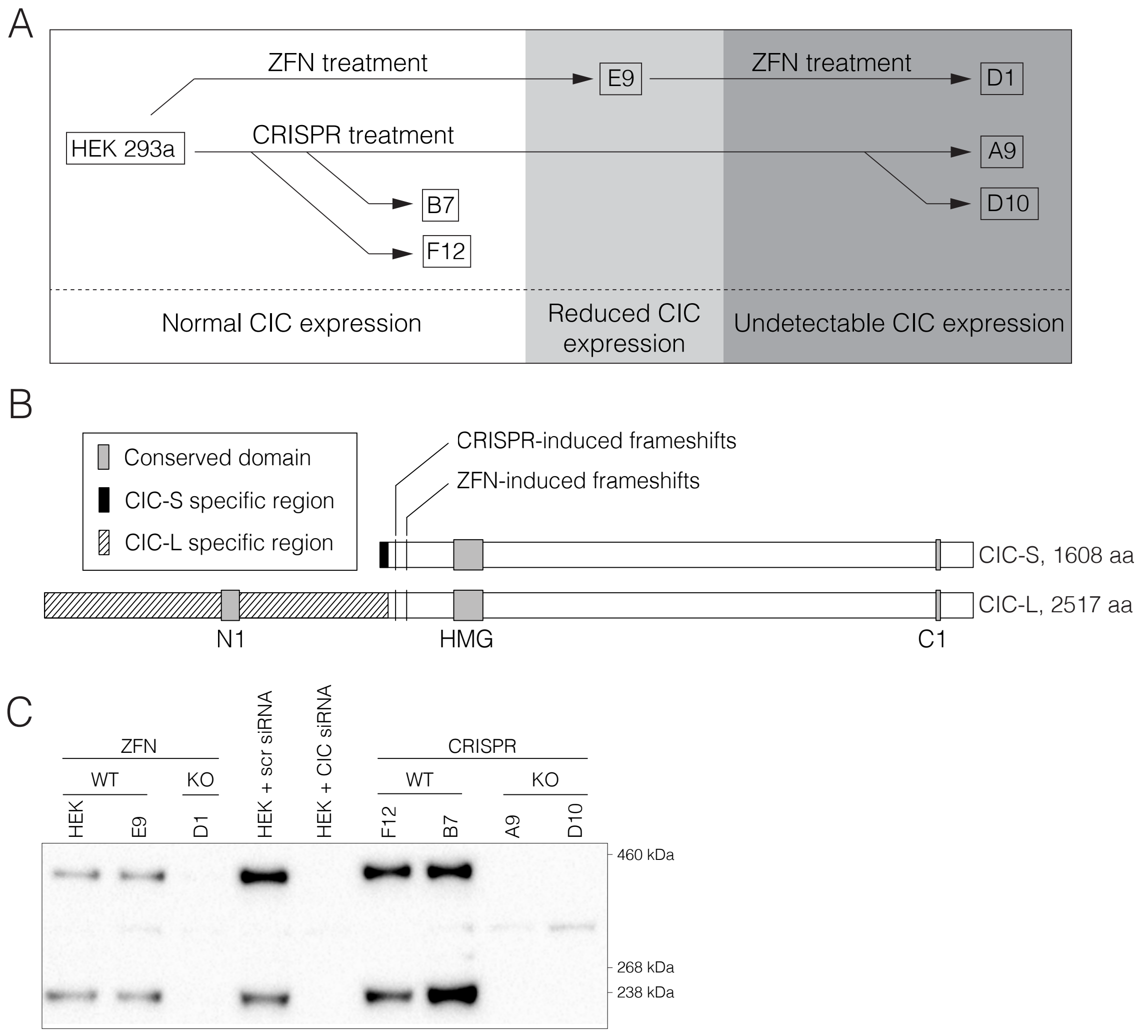

Figure S2. Generation of *CIC* knockout cell lines. (**A**) Scheme illustrating the generation of *CIC* knockout cell lines using the ZFN and CRISPR/Cas9 systems. (**B**) Protein structure of the CIC isoforms (short [CIC-S] and long [CIC-L]) annotated with conserved domains. N1: conserved N-terminal domain. HMG: DNA-binding high mobility group box domain. C1: conserved C-terminal domain. (**C**) Additional Western blot showing lack of CIC expression in *CIC* knockout cell lines (see Fig 1A).
