# Supplementary material for: Comparative transcriptome analysis of isogenic cell line models and primary cancers links capicua (CIC) loss to activation of the MAPK signalling cascade
Source: J Pathol. 2017 Apr 26;242(2):206–20. doi: 10.1002/path.4894 (PMC5485162; doi:10.1002/path.4894)
Supplement: Supplementary file 5 — Figure S3. CIC missense mutants retain repressive activity. (A) Representative Western blot of cells used for the luciferase assays. D1 cells were transfected with the indicated constructs, and TBP was used as a loading control. R201W and R1515H are missense mutations in the HMG and C1 domains, respectively. Q564X is a nonsense mutation that results in a truncated form of CIC. (B) Diagram of the relevant portion of the luciferase reporter construct used. The numbers represent distance (in bp) from the ETV5 transcription start site. (C) Relative luciferase expression in cells transfected with indicated CIC‐S constructs. Loss of CIC‐mediated repression is clear in the Q564X nonsense mutation, while the missense mutants retain repressive activity similar to the wild type construct. Error bars: s.e.m. over three independent experiments. *p < 0.05, **p < 0.01, ***p < 0.001 compared to the vector‐only control (two‐sided Student's t‐test). [file PATH-242-206-s005.pdf]

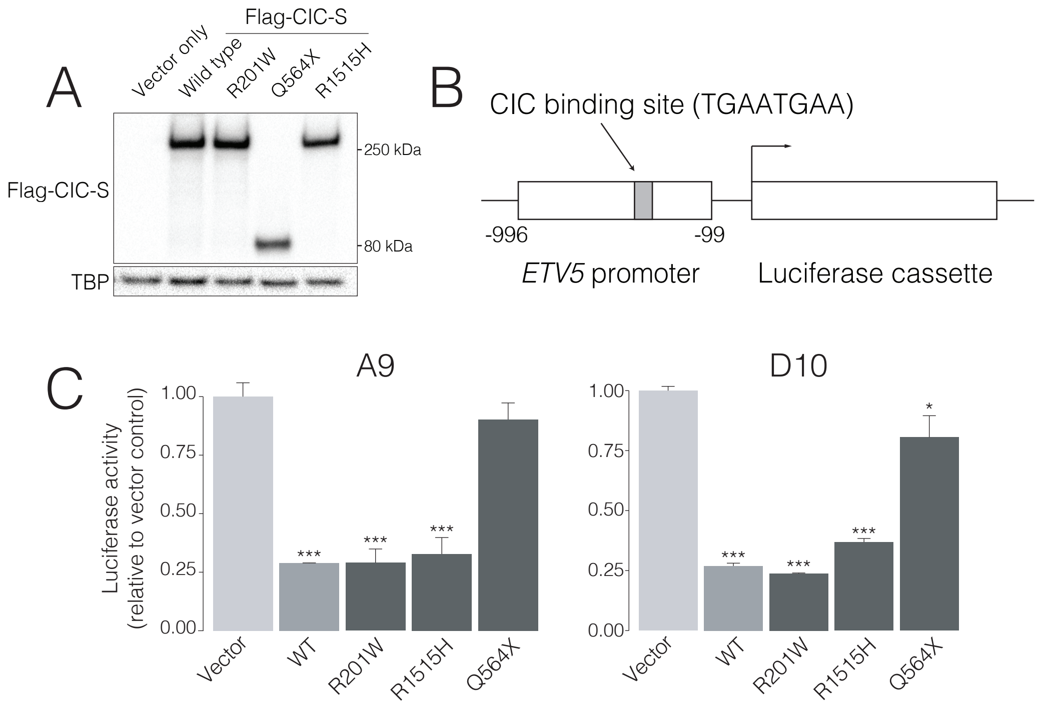

Figure S3. *CIC* missense mutants retain repressive activity. (**A**) Representative western blot of cells used for the luciferase assays. D1 cells were transfected with the indicated constructs, and TBP was used as a loading control. R201W and R1515H are missense mutations in the HMG and C1 domains, respectively. Q564X is a nonsense mutation that results in a truncated form of CIC. (**B**) Diagram of the relevant portion of the luciferase reporter construct used. The numbers represent distance (in bp) from the *ETV5* transcription start site. (**C**) Relative luciferase expression in cells transfected with indicated *CIC* constructs. Loss of CIC-mediated repression is clear in the Q564X nonsense mutation, while the missense mutants retain repressive activity similar to the wild type construct. Error bars: s.e.m. over three independent experiments. *p < 0.05, **p < 0.01, ***p < 0.001 compared to the vector-only control (two-sided Student's *t*-test).
