# Supplementary material for: Comparative transcriptome analysis of isogenic cell line models and primary cancers links capicua (CIC) loss to activation of the MAPK signalling cascade
Source: J Pathol. 2017 Apr 26;242(2):206–20. doi: 10.1002/path.4894 (PMC5485162; doi:10.1002/path.4894)
Supplement: Supplementary file 6 — Figure S4. ETV4 shows increased protein expression in CICKO cell lines. Quantification for Western blot shown in Figure 3B. Error bars: s.e.m. over three independent experiments. [file PATH-242-206-s006.pdf]

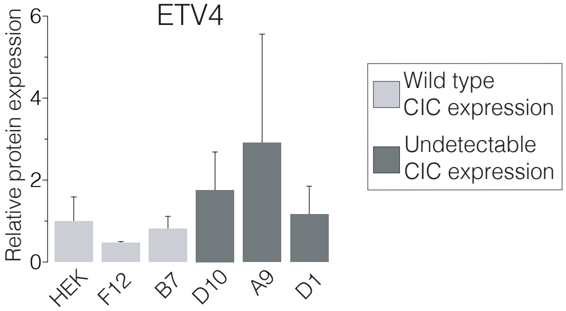

Figure S4. ETV4 shows increased protein expression in *CIC*^{KO} cell lines. Quantification for Western blot shown in Fig 3B. Error bars: s.e.m. over three independent experiments.
