# Supplementary material for: Comparative transcriptome analysis of isogenic cell line models and primary cancers links capicua (CIC) loss to activation of the MAPK signalling cascade
Source: J Pathol. 2017 Apr 26;242(2):206–20. doi: 10.1002/path.4894 (PMC5485162; doi:10.1002/path.4894)
Supplement: Supplementary file 7 — Figure S5. Targeted ChIP‐qPCR analysis of high‐confidence candidate targets of CIC. Zoomed‐in views of Figure 4B for each putative CIC binding site tested. Isoforms were obtained from the UCSC genome browser (Hg38), and respective IDs are shown. Chromosomal locations are also indicated. The sequence found within each site is indicated, with mismatches underlined. Bar plots show relative enrichment of each site compared to NCR1 in CIC WT samples (light grey) and CIC KO samples (dark grey). NCR1 and NCR2 (not shown) are located ∼1 kb upstream of ETV4 Site A and ∼1 kb downstream of ETV4 Site C, respectively. Red and blue bars indicate sites found on the positive and negative strands, respectively. Error bars: s.d. over four (WT) or three (KO) independent experiments. *p < 0.05, **p < 0.01, ***p < 0.001 [file PATH-242-206-s007.pdf]

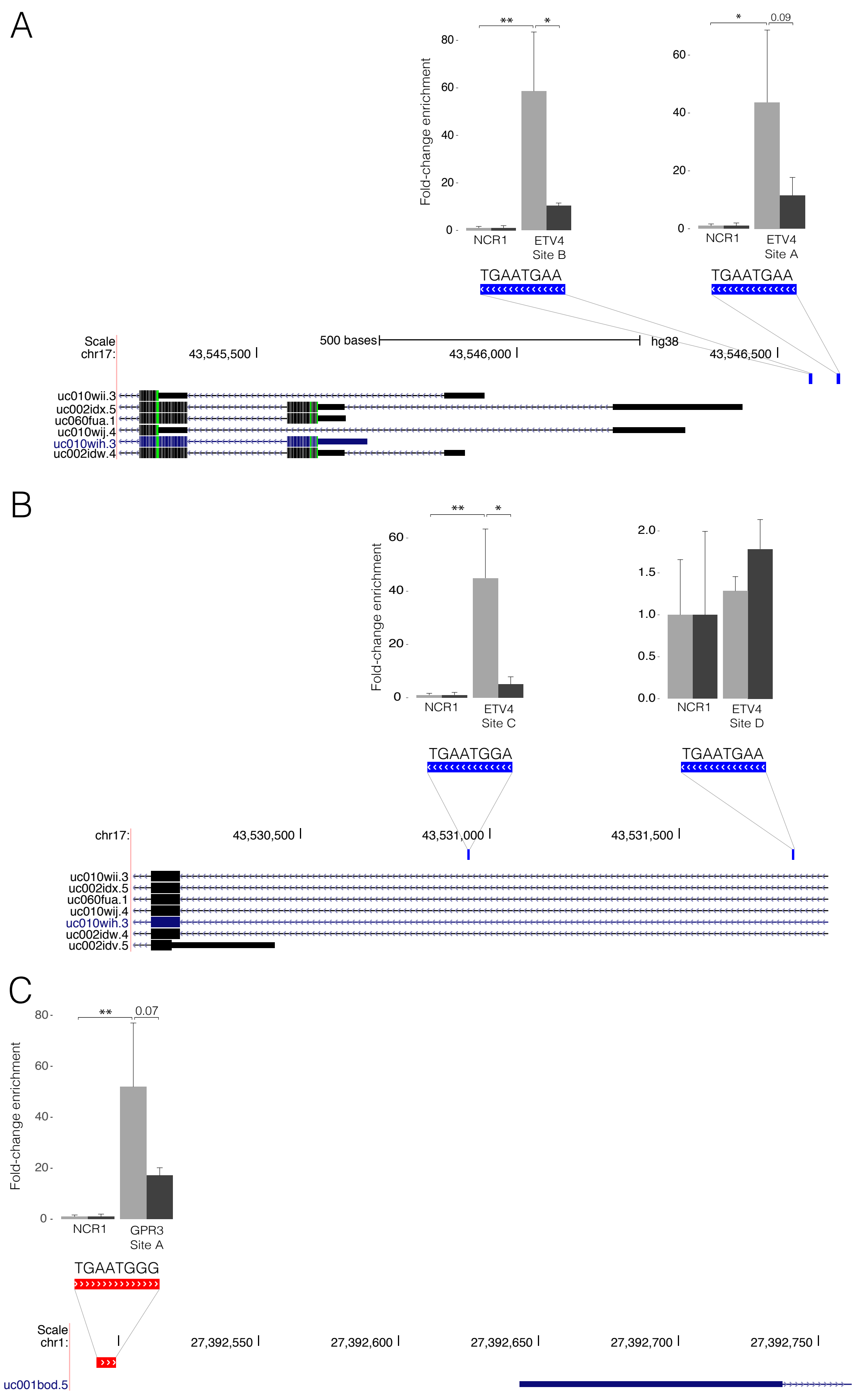
A
Fold-change enrichment
** *
80
60
40
20
0
NCR1
ETV4
Site B
* 0.09
60
40
20
0
NCR1
ETV4
Site A
TGAATGAA
TGAATGAA
Scale
chr17:
500 bases
hg38
43,545,500
43,546,000
43,546,500
uc010wii.3
uc002idx.5
uc060fua.1
uc010wij.4
uc010wih.3
uc002idw.4
B
Fold-change enrichment
** *
60
40
20
0
NCR1
ETV4
Site C
2.0
1.5
1.0
0.5
0.0
NCR1
ETV4
Site D
TGAATGGA
TGAATGAA
chr17:
43,530,500
43,531,000
43,531,500
uc010wii.3
uc002idx.5
uc060fua.1
uc010wij.4
uc010wih.3
uc002idw.4
uc002idv.5
C
Fold-change enrichment
** 0.07
80
60
40
20
0
NCR1
GPR3
Site A
TGAATGGG
Scale
chr1:
27,392,550
27,392,600
27,392,650
27,392,700
27,392,750
uc001bod.5

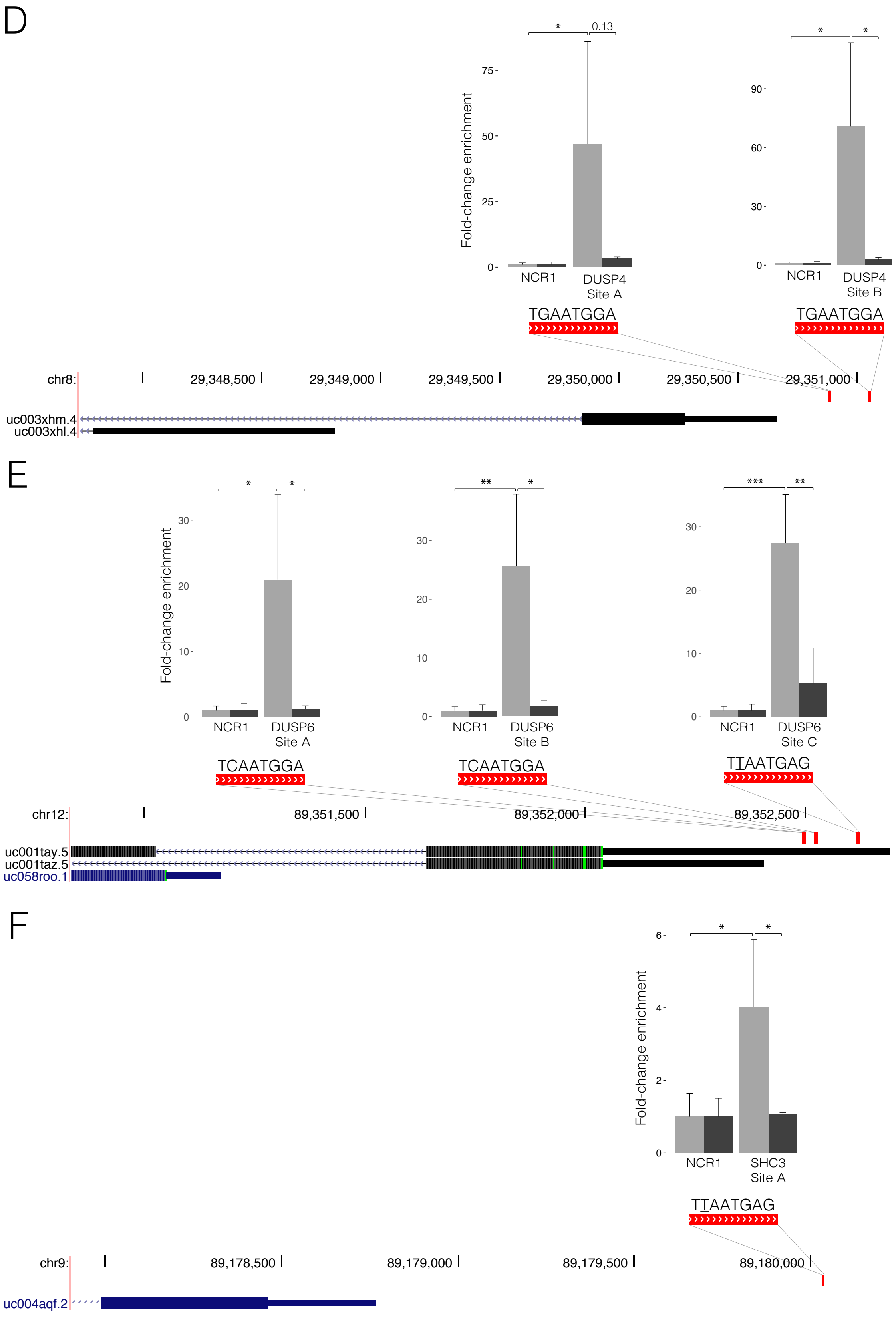
D
* 0.13
* *
Fold-change enrichment
NCR1
DUSP4 Site A
DUSP4 Site B
TGAATGGA
chr8:
29,348,500
29,349,000
29,349,500
29,350,000
29,350,500
29,351,000
uc003xhm.4
uc003xhl.4
E
Fold-change enrichment
NCR1
DUSP6 Site A
DUSP6 Site B
DUSP6 Site C
TCAATGGA
TTAATGAG
chr12:
89,351,500
89,352,000
89,352,500
uc001tay.5
uc001taz.5
uc058roo.1
F
Fold-change enrichment
NCR1
SHC3 Site A
TTAATGAG
chr9:
89,178,500
89,179,000
89,179,500
89,180,000
uc004aqf.2

G

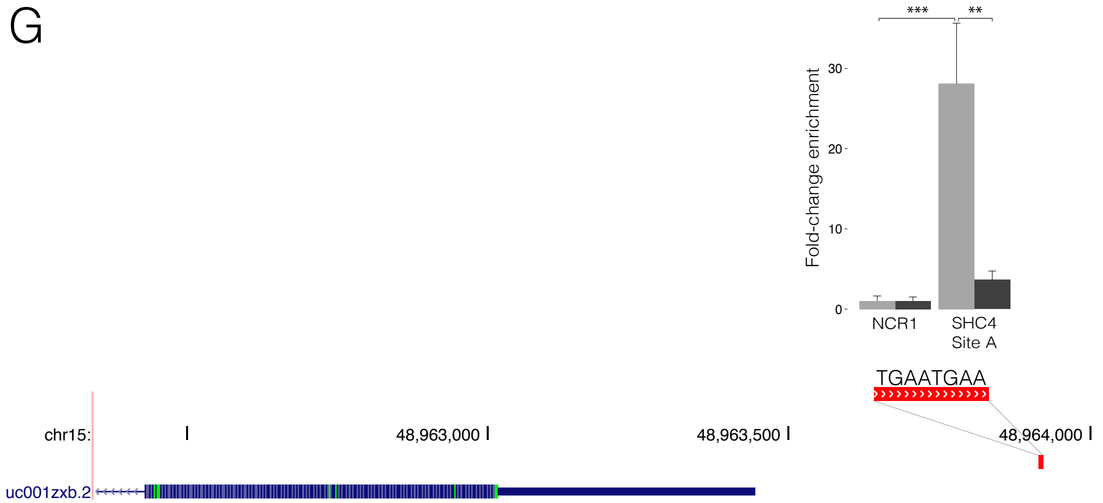

***
**
Fold-change enrichment
30
20
10
0
NCR1
SHC4
Site A
TGAATGAA
chr15:
48,963,000
48,963,500
48,964,000
uc001zxb.2

H

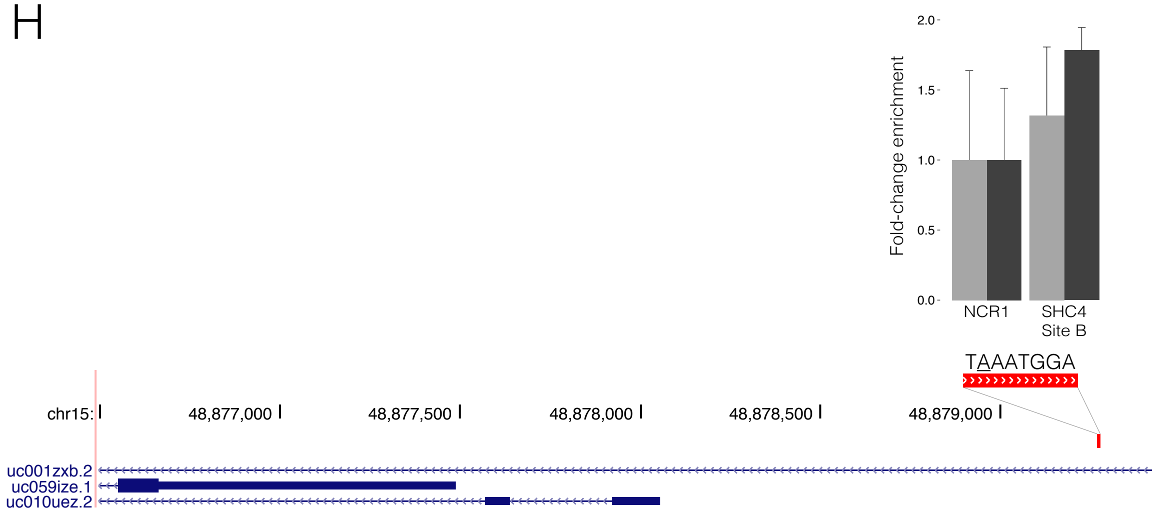

Fold-change enrichment
2.0
1.5
1.0
0.5
0.0
NCR1
SHC4
Site B
TAAATGGA
chr15:
48,877,000
48,877,500
48,878,000
48,878,500
48,879,000
uc001zxb.2
uc059ize.1
uc010uez.2

I

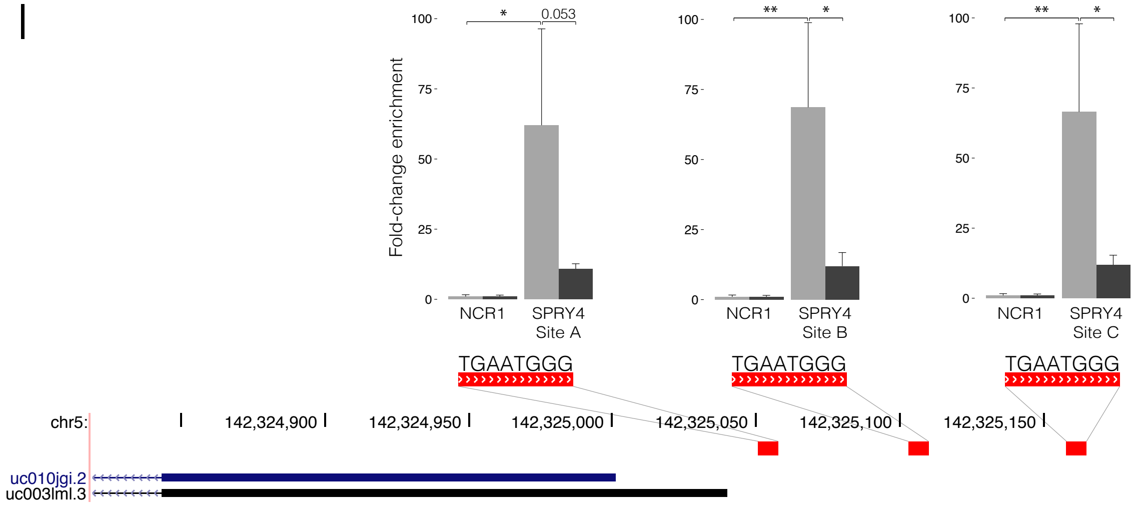

Fold-change enrichment
100
75
50
25
0
*
0.053
**
*
**
*
NCR1
SPRY4
Site A
NCR1
SPRY4
Site B
NCR1
SPRY4
Site C
TGAATGGG
TGAATGGG
TGAATGGG
chr5:
142,324,900
142,324,950
142,325,000
142,325,050
142,325,100
142,325,150
uc010jgi.2
uc003lml.3

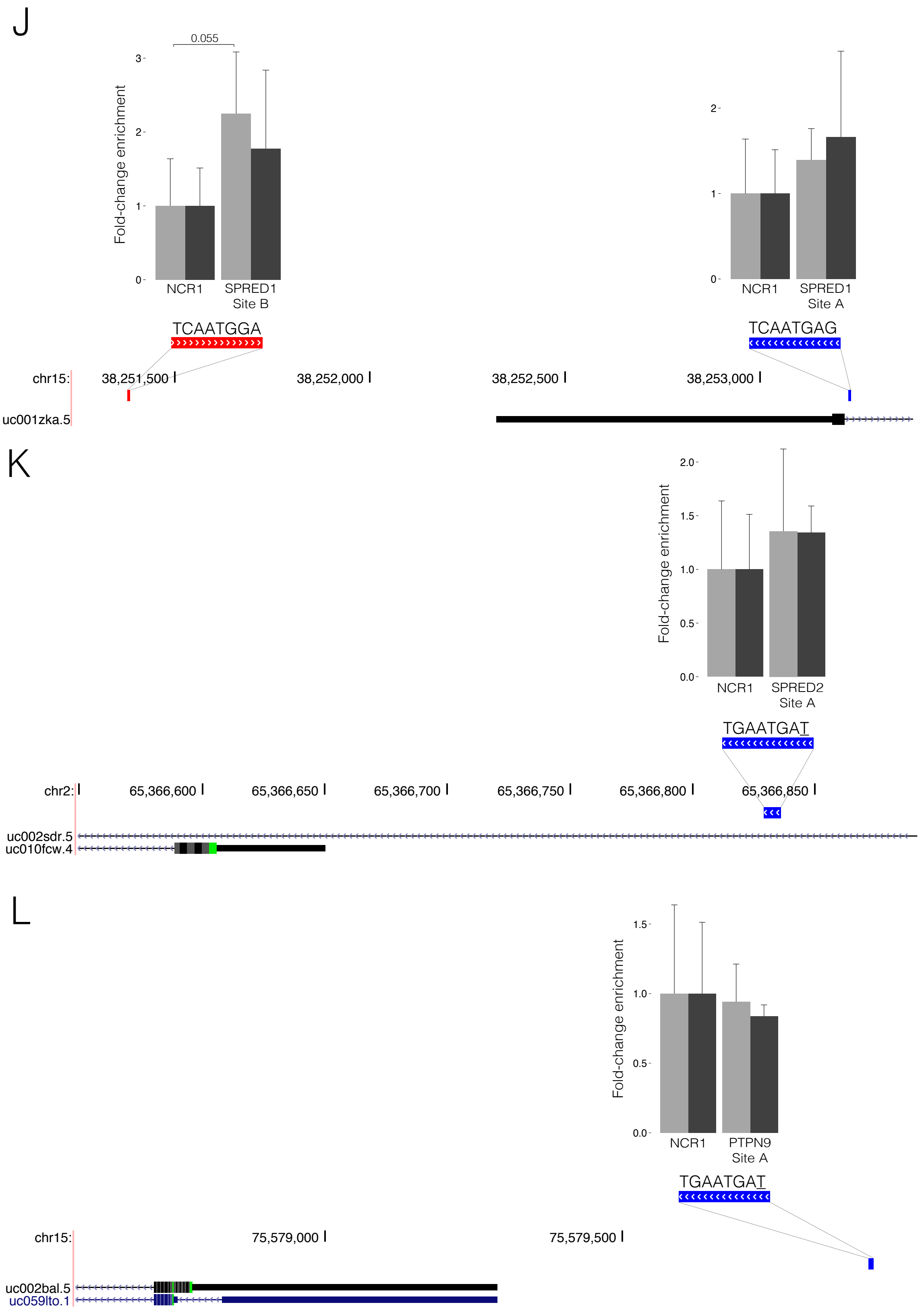
J
0.055
Fold-change enrichment
NCR1
SPRED1
Site B
TCAATGGA
NCR1
SPRED1
Site A
TCAATGAG
chr15:
38,251,500
38,252,000
38,252,500
38,253,000
uc001zka.5
K
Fold-change enrichment
NCR1
SPRED2
Site A
TGAATGAT
chr2:
65,366,600
65,366,650
65,366,700
65,366,750
65,366,800
65,366,850
uc002sdr.5
uc010fcw.4
L
Fold-change enrichment
NCR1
PTPN9
Site A
TGAATGAT
chr15:
75,579,000
75,579,500
uc002bal.5
uc059lto.1

Figure S5. Targeted ChIP-qPCR analysis of high-confidence candidate targets of CIC. Zoomed-in views of Fig 4B for each putative CIC binding site tested. Isoforms were obtained from the UCSC genome browser (Hg38), and respective IDs are shown. Chromosomal locations are also indicated. The sequence found within each site is indicated, with mismatches underlined. Bar plots show relative enrichment of each site compared to NCR1 in CIC^{WT} samples (light grey) and CIC^{KO} samples (dark grey). NCR1 and NCR2 (not shown) are located ~1kb upstream of *ETV4* Site A and ~1kb downstream of *ETV4* Site C, respectively. Red and blue bars indicate sites found on the positive and negative strands, respectively. Error bars: s.d. over four (WT) or three (KO) independent experiments. *$p < 0.05$, **$p < 0.01$, ***$p < 0.001$
