# Supplementary material for: Comparative transcriptome analysis of isogenic cell line models and primary cancers links capicua (CIC) loss to activation of the MAPK signalling cascade
Source: J Pathol. 2017 Apr 26;242(2):206–20. doi: 10.1002/path.4894 (PMC5485162; doi:10.1002/path.4894)
Supplement: Supplementary file 8 — Figure S6. CIC loss leads to increased expression of downstream MAPK targets. (A) UpSet plot showing overlap of DE genes in the four contexts we studied. (B) Additional quantifications for Western blots shown in Figure 5C, shown relative to HEK + scr siRNA . Error bars: s.e.m. over three independent experiments. *p < 0.05, **p < 0.01 (two‐sided Student's t‐test). (C) Representative Western blots of indicated cell lines treated with a vehicle control (DMSO) or a MEK inhibitor (Trametinib). Results from this treatment are consistent with results seen following MEK/ERK knockdown using siRNAs (Figure 5C). Tubulin was used as a loading control, and a representative blot is shown. [file PATH-242-206-s008.pdf]

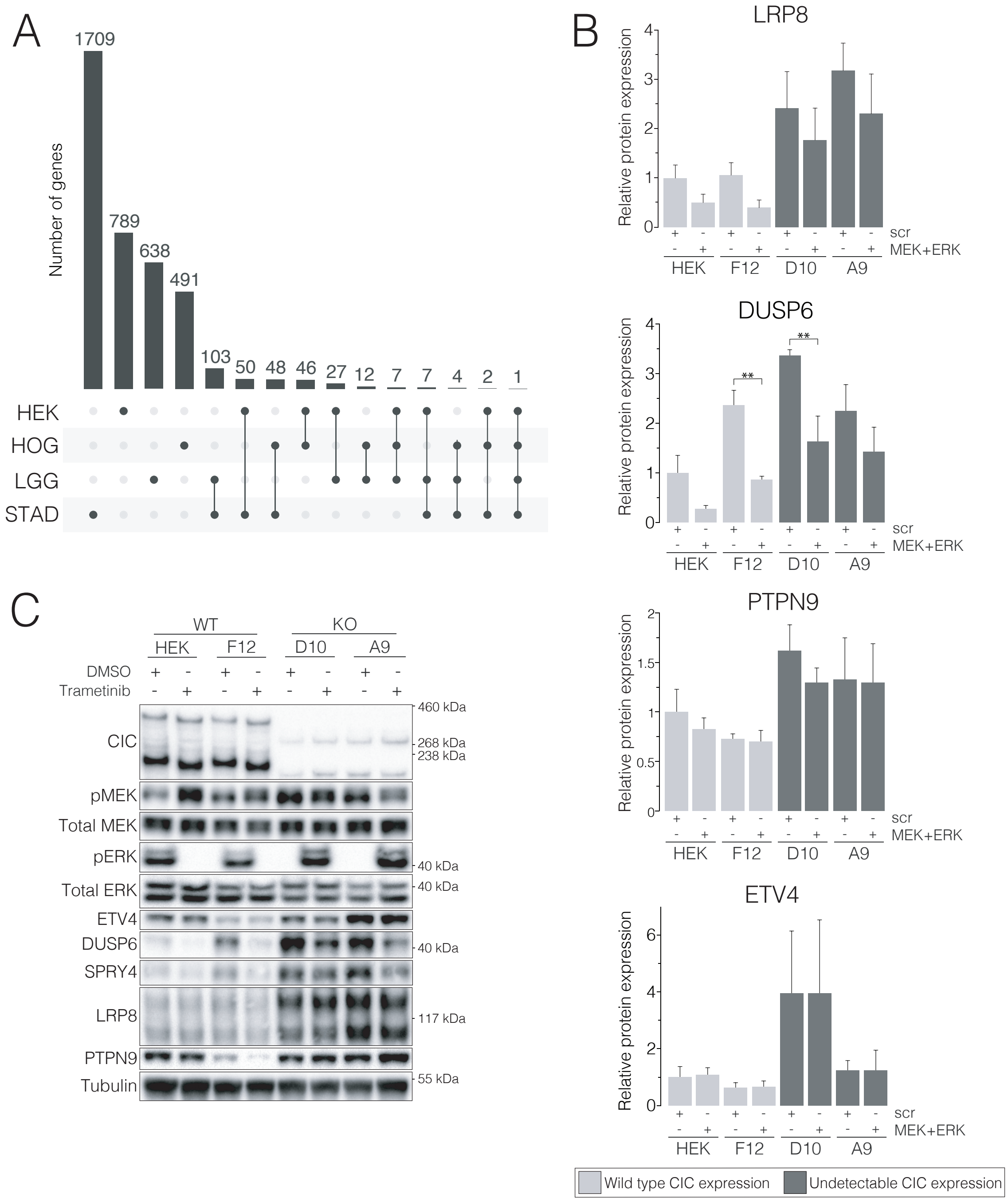

Figure S6. CIC loss leads to increased expression of downstream MAPK targets. (**A**) UpSet plot showing overlap of DE genes in the four contexts studied. (**B**) Additional quantifications for western blots shown in Fig 5C, shown relative to HEK + scr siRNA . Error bars: s.e.m. over three independent experiments. *p < 0.05, **p < 0.01 (two-sided Student's *t*-test). (**C**) Representative Western blots of indicated cell lines treated with a vehicle control (DMSO) or a MEK inhibitor (Trametinib). Results from this treatment are consistent with results seen following *MEK*/*ERK* knockdown using siRNAs (Fig 5C). Tubulin was used as a loading control, and a representative blot is shown.
